# Selective Synthesis of Bismuth or Bismuth Selenide Nanosheets from a Metal Organic Precursor: Investigation of their Catalytic Performance for Water Splitting

**DOI:** 10.1021/acs.inorgchem.0c02668

**Published:** 2021-01-19

**Authors:** Shumaila Razzaque, Malik Dilshad Khan, Muhammad Aamir, Manzar Sohail, Sanket Bhoyate, Ram K. Gupta, Muhammad Sher, Javeed Akhtar, Neerish Revaprasadu

**Affiliations:** † Key Laboratory of Material Chemistry for Energy Conversion and Storage, Ministry of Education, School of Chemistry and Chemical Engineering, Huazhong University of Science and Technology, Luoyu Road No. 1037, Wuhan 430074, China; ‡ Institute of Physical Chemistry, Polish Academy of Sciences, Kasprzaka 44/52, 01-224 Warsaw, Poland; § Department of Chemistry, University of Zululand, Private Bag X1001, Kwa-Dlangezwa 3880, South Africa; ∥ Department of Chemistry, Materials Laboratory, Mirpur University of Science & Technology (MUST), Mirpur 10250, AJK, Pakistan; ⊥ Department of Chemistry, School of Natural Sciences, National University of Science and Technology, H-12, Islamabad 46000, Pakistan; # Department of Chemistry, Pittsburg State University, Pittsburg, Kansas 66762, United States; ∇ Department of Chemistry, Allama Iqbal Open University, Islamabad 44000, Pakistan

## Abstract

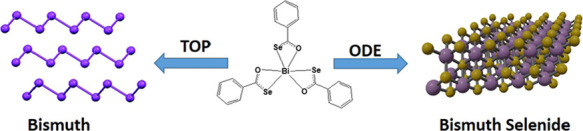

The development of cost-effective, functional materials that can be efficiently used for sustainable energy generation is highly desirable. Herein, a new molecular precursor of bismuth (tris(selenobenzoato)bismuth(III), [Bi(SeOCPh)_3_]), has been used to prepare selectively Bi or Bi_2_Se_3_ nanosheets via a colloidal route by the judicious control of the reaction parameters. The Bi formation mechanism was investigated, and it was observed that the trioctylphosphine (TOP) plays a crucial role in the formation of Bi. Employing the vapor deposition method resulted in the formation of exclusively Bi_2_Se_3_ films at different temperatures. The synthesized nanomaterials and films were characterized by p-XRD, TEM, Raman, SEM, EDX, AFM, XPS, and UV–vis spectroscopy. A minimum sheet thickness of 3.6 nm (i.e., a thickness of 8–9 layers) was observed for bismuth, whereas a thickness of 4 nm (i.e., a thickness of 4 layers) was observed for Bi_2_Se_3_ nanosheets. XPS showed surface oxidation of both materials and indicated an uncapped surface of Bi, whereas Bi_2_Se_3_ had a capping layer of oleylamine, resulting in reduced surface oxidation. The potential of Bi and Bi_2_Se_3_ nanosheets was tested for overall water-splitting application. The OER and HER catalytic performances of Bi_2_Se_3_ indicate overpotentials of 385 mV at 10 mA cm^–2^ and 220 mV, with Tafel slopes of 122 and 178 mV dec^–1^, respectively. In comparison, Bi showed a much lower OER activity (506 mV at 10 mA cm^–2^) but a slightly better HER (214 mV at 10 mA cm^–2^) performance. Similarly, Bi_2_Se_3_ nanosheets were observed to exhibit cathodic photocurrent in photoelectrocatalytic activity, which indicated their p-type behavior.

## Abstract 2

Bismuth selenobenzoate has been used for selective synthesis of bismuth or bismuth selenide nanosheets, by judicious control of reaction parameters. Electrocatalytic water splitting performance of bismuth and bismuth selenide nanosheets is also investigated.

## Introduction

Layered materials, when thinned down to their atomic limits, are referred to as two-dimensional (2D) materials that exhibit unique properties as compared to their bulk counterparts. The thinning of the bulk layered material may result in enhanced mechanical,^1^ conductive,^2^ and optoelectronic properties,^3^ which differ from the parent material. Among the layered materials, the remarkable improvement in properties of graphene has encouraged researchers to explore other 2D materials, which also show interesting and exceptional properties.^4^ Furthermore, the availability of layered materials in different oxidation states and the type of chalcogenide (S, Se, or Te) provide more flexibility in the optimization of the desired properties.

Recently, the ever-increasing energy demand has shifted the research focus toward the development of suitable nanomaterials for energy harvesting. The use of hydrogen as an alternative green fuel is a viable option; however, obtaining hydrogen in large quantities is a challenge. Water is the cheapest source of hydrogen; however, the process of water splitting to obtain hydrogen requires suitable catalysts as the process is thermodynamically not feasible. The use of cost-effective nanomaterials for efficient water splitting by evading expensive metals, i.e., ruthenium, gold, or platinum, is necessary for industrial applications. Two-dimensional materials have shown promising applications for renewable energy conversion devices and are potential candidates to address the energy crisis.^5^

Bismuth, from the pnictogen family, is of particular interest as it shows a layered structure in both unary and binary forms (i.e., bismuthene or Bi_2_Se_3_). Both Bi and Bi_2_Se_3_ exist in the rhombohedral crystal structure, with band gaps varying between 0.35 and 0.99 eV, depending on the thickness of the sheets.^6^ Bismuth has been considered as a potential material for energy storage and catalysis applications because of its high environmental stability and unique electronic properties. It shows a high theoretical volumetric capacity of 3765 mAh cm^–3^ due to its high density, which makes it a highly suitable anodic material replacing other well-investigated 2D materials, such as black phosphorus (2266 mAh cm^–3^) and graphite (837 mAh cm^–3^).^7^ Similarly, the narrow energy gap and the layered structure of Bi_2_Se_3_ make it a suitable material for high-performance infrared detectors and thermoelectric applications,^8^ and it has recently been demonstrated to be a reference three-dimensional topological insulator,^9^ with an insulating bulk gap of 0.3 eV and metallic surface states consisting of a single Dirac cone.^10^ The unusual surface of Bi_2_Se_3_ exhibits an unconventional spin texture, electron dynamics, and stability characteristic.^9b^ It has great potential to serve as a conductive substrate to enhance HER and supercapacitor performances.^11^ Furthermore, the layered structure of Bi_2_Se_3_ allows easy intercalation between layers, offering a high conducting and charging lane in electrode materials.

Various synthetic approaches have been developed for the synthesis of these materials.^12−16^ Bi_2_Se_3_ nanosheets/nanodiscs have been synthesized by the multisource colloidal route,^12^ microwave synthesis,^13^ solvothermal/ionothermal route,^14^ and exfoliation.^15^ On the other hand, bismuth nanomaterials are usually prepared by the reduction of different bismuth salts by employing different reducing agents/conditions or by exfoliation.^16^ However, there is no report of the preparation of bismuth from metal–organic precursors, and only a few such complexes have been used so far for the synthesis of Bi_2_Se_3_ nanosheets. The molecular precursor route is desirable because the presence of preformed bonds between the metal atom and the chalcogenide atom in single-source precursors often provides better control over size and morphology.^17^ The only complexes available to date to prepare Bi_2_Se_3_ nanosheets and/or films are 2-pyridyl selenolates,^18^ diselenoimidophosphinate,^19^ dialkyldiseleno phosphate,^20^ and dialkyldiselenocarbamate complexes.^21^ However, molecular precursors containing phosphorus may cause phosphorus contamination in the final product or formation of a phosphate product exclusively.^20^ Similarly, the synthesis of diselenocarbamate precursors involves the use of highly unstable, obnoxious, and expensive CSe_2_, which is commercially unavailable and very difficult to synthesize.^22^

The colloidal method using metal–organic precursors provides easy control over reaction parameters and better reproducibility. A range of options are available for selecting different capping agents such as primary amines, carboxylic acids, and thiols, and highly crystalline products can be achieved in a short duration of time. Since a single starting material is being used here for the preparation of Bi or Bi_2_Se_3_ nanosheets and thin films, the flexibility of the starting material makes the process facile and comparatively economical. The metal–organic complexes are generally air- and moisture-stable, which makes their handling and storage easy. Furthermore, the mild reaction conditions offer a range of temperatures at which reactions can be performed without requiring state-of-the-art equipment or stringent requirements. Herein, the synthesis of a new selenium-based complex of bismuth, i.e., tris(selenobenzoato)bismuth(III) [Bi(SeOCPh)_3_], is reported by a facile route. The synthesized complex is versatile as it was successfully employed for the preparation of bismuth or bismuth selenide nanosheets by a solvothermal route, whereas the decomposition of the precursor by the vapor deposition method yielded Bi_2_Se_3_ films. The colloidally synthesized Bi and Bi_2_Se_3_ nanosheets were also tested for electrocatalytic water splitting.

## Experimental Section

### Materials

All reagents, i.e., BiCl_3_, NaBH_4_, C_6_H_5_COCl, Se, 1-octadecene (ODE), oleylamine (OLA), trioctylphosphine (TOP), ethanol, acetone, and THF, were obtained from Sigma Aldrich.

### Synthesis of [Bi(SeOCPh)_3_] Complex

**Scheme 1:**
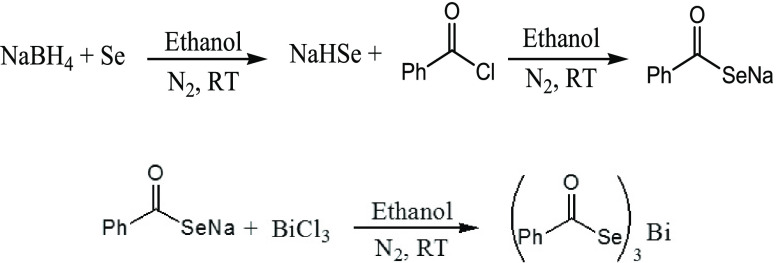
Preparation of the Selenobenzoate Ligand and the Tris(selenobenzoato)Bi(III) Complex

NaHSe was synthesized by treating 6.0 mmol of elemental Se powder with 6.0 mmol of NaBH_4_ in ethanol (50.0 mL) under a nitrogen atmosphere at room temperature. The decolorization of the solution from reddish to colorless within 8–10 min of stirring indicates the generation of NaHSe. After a further 15 min of stirring, benzoyl chloride in equimolar quantity was injected slowly into freshly prepared NaHSe solution. An immediate appearance of yellow color indicates the generation of the selenobenzoate ligand. After the complete addition of acid chloride, the reaction mixture was stirred continuously for another half an hour to ensure completion of the reaction. After half an hour of stirring, BiCl_3_ (0.48 g, 2.0 mmol) dissolved in ethanol (20.0 mL) was slowly added while stirring. The reaction was continued for approximately 30 min, after which the formed precipitate was filtered and washed with an excess of ethanol. The schematic reaction for the synthesis of the selenobenzoate complex of bismuth is shown in Scheme 1. The recrystallization of the precipitate, using THF, yielded a yellowish powder. Melting point: 177–178 °C; elemental analysis calcd (%) for elemental analysis for C_21_H_15_O_3_BiSe_3_, found: C 33.39, H 1.97, Bi 27.18; calcd (%): C 33.17, H 1.99, Bi 27.45.

### Synthesis of Bi_2_Se_3_ Nanosheets

Oleylamine (8.0 mL) was heated to 200 °C under a nitrogen environment. Once the desired temperature was reached, the tris(selenobenzoato)bismuth(III) complex (0.40 mmol), dispersed in ODE (3.0 mL), was injected into preheated OLA under vigorous stirring. A decrease in temperature (≈15–20 °C) was noted upon injection, and the solution instantly turned brownish-black. The temperature was quickly readjusted to 200 °C, where it was maintained for 1 h, after which the heating source was removed and the solution cooled to room temperature. A 40.0 mL mixture of acetone and methanol (1:1) was added to the cooled solution, and the precipitated Bi_2_Se_3_ nanosheets were washed and separated by centrifugation.

### Synthesis of Bismuth

Bismuth was prepared by following a similar procedure, as used for the synthesis of Bi_2_Se_3_ nanosheets, except that the precursor was dispersed in trioctylphosphine (TOP) instead of ODE. It was noted that the color of the complex starts to change when the complex was dispersed in TOP. The dispersed complex was immediately injected into the preheated OLA (at 200 °C), and the reaction was continued for 1 h. There was no dispersion of particles; instead, the particles flocculate immediately, forming a solid chunk that precipitated out of the solution. The precipitate was washed and separated by centrifugation using an acetone and methanol (1:1) mixture. The solid chunk was crushed with a spatula to a powdered form, which was then used for further analysis.

### Deposition of Bi_2_Se_3_ Thin Films

Bismuth selenide films were deposited on glass substrates. The surface of glass substrates was cleaned by sonicating in HNO_3_, washed with deionized water, and then rinsed in acetone. The AACVD setup consists of an ultrasonic humidifier (to generate aerosol) and a carbolite tube furnace. For the deposition of thin films, a two-necked round-bottom flask (RBF) containing a freshly prepared solution of tris(selenobenzoato)bismuth(III) (0.3 mmol) in chloroform (20.0 mL) was placed over the humidifier. The reactor tube, containing eight glass substrates, was inserted into the tube furnace. Reinforced tubings were used to connect the gas inlet to one neck of the RBF and the reactor tube by the other neck. The ultrasonic humidifier generated the aerosol, which was then transferred to the heating zone of the furnace by argon. The flow rate was adjusted to 180 sccm, and the deposition of Bi_2_Se_3_ films took place inside the heating chamber on the surface of the heated glass substrates.

### Characterization

Elemental analysis was performed using a Thermo Scientific Flash 2000 Organic Elemental Analyzer. The decomposition behavior of the precursor was observed by a Mettler-Toledo thermogravimetric system. P-XRD was obtained from a Bruker D8 Discover Diffractometer. A Talos F200X microscope, operating at 200 kV and equipped with an FEI ceta camera, was used to capture TEM and HRTEM images. Carbon coating of thin films was performed on an Edwards coating system E306A. Scanning electron microscopic (SEM) images and EDX analyses were obtained from Philips XL30 FEG SEM. Raman analysis was performed using a Renishaw 1000 Micro-Raman System, and PerkinElmer Lambda 1050 instrument was used for the UV–vis–NIR spectrum.

## Results and Discussions

Among the monochalcogeno-carboxylic acids, various complexes of thiocarboxylic acid have been reported, but there are only a few reports of the congeners containing heavier chalcogenide atoms (i.e., Se or Te), probably due to their instability and the handling difficulties associated with them. Selenobenzoic acid is highly sensitive and cannot be isolated as it is easily oxidized to its dimer, i.e., dibenzoyl diselenide. However, it can be stabilized by the formation of a salt or in solution under inert conditions. In the present study, the bismuth–selenobenzoate complex was prepared by a new method, which is comparatively more efficient than a previously reported method.^23^ The previous method required a reaction between an alkali metal (M = Na, K) and Se to prepare M_2_Se, in ammonia at −70 °C for 12 h.^24^ Our synthetic protocol can be performed at room temperature in ethanol. NaBH_4_ replaced the highly pyrophoric alkali metals. Moreover, the reaction can be completed within a short duration of time, i.e., almost in half an hour. Elemental analysis and TGA were used to characterize the final product. Repeated attempts to recrystallize this product to determine the X-ray crystal structure were unsuccessful as the product starts to decompose.

The decomposition behavior of the complex was analyzed by TGA (Figure S1, SI). The decomposition of the precursor took place in three steps, and the precursor indicates high thermal stability in the solid state. A substantial weight loss (almost ∼37%) was detected in the first step, in the temperature range of 160–240 °C, which, possibly, shows the loss of C_6_H_5_ moieties. A further 12% decrease in mass was detected in the second step, which infers exclusion of the carbonyl moiety, leaving behind the BiSe_3_ unit. Finally, two such fragments undergo a transformation from BiSe_3_ to Bi_2_Se_3_ with a little loss of Se (9% weight loss) at high temperature, and schematically it is shown in Scheme S1.^25^ The complex decomposes completely around ≈385 °C, and a final residue of ∼42% was obtained, which corresponds to the formation of Bi_2_Se_3_ (theoretically ca. 43%).

### Bi_2_Se_3_ Nanosheets

The bismuth selenide nanosheets were synthesized at 200 °C by decomposition of tris(selenobenzoato)bismuth(III) (dispersed in ODE) in OLA. The reaction mixture turned brownish-black immediately upon injecting the complex in oleylamine, which may indicate the formation of nanomaterials. Similarly, it was observed that the addition of the complex in OLA, even at room temperature, starts changing the color of the solution to brownish-black, indicating the decomposition of the complex; however, the product formed was amorphous. Hence, to obtain Bi_2_Se_3_ nanosheets with reasonably high crystallinity, a reaction temperature of 200 °C was used. It shows that OLA, besides acting as a surface-passivating agent, also initiates the degradation of the precursors. The degradation of the selenobenzoate complexes in the presence of primary amines may undergo a similar decomposition pathway, as indicated by Chin et al. for primary amine-assisted decomposition of thiocarboxylate complexes.^26^ Previously, decomposition of the silver selenobenzoate was observed even at room temperature in oleylamine by Vittal and co-workers.^27^ The decomposition is accelerated by the primary amine, which acts as a nucleation initiator as well. In this way, the nucleation and the growth steps are separated, which is preferable to produce monodispersed nanomaterials.^28^

**Figure 1:**
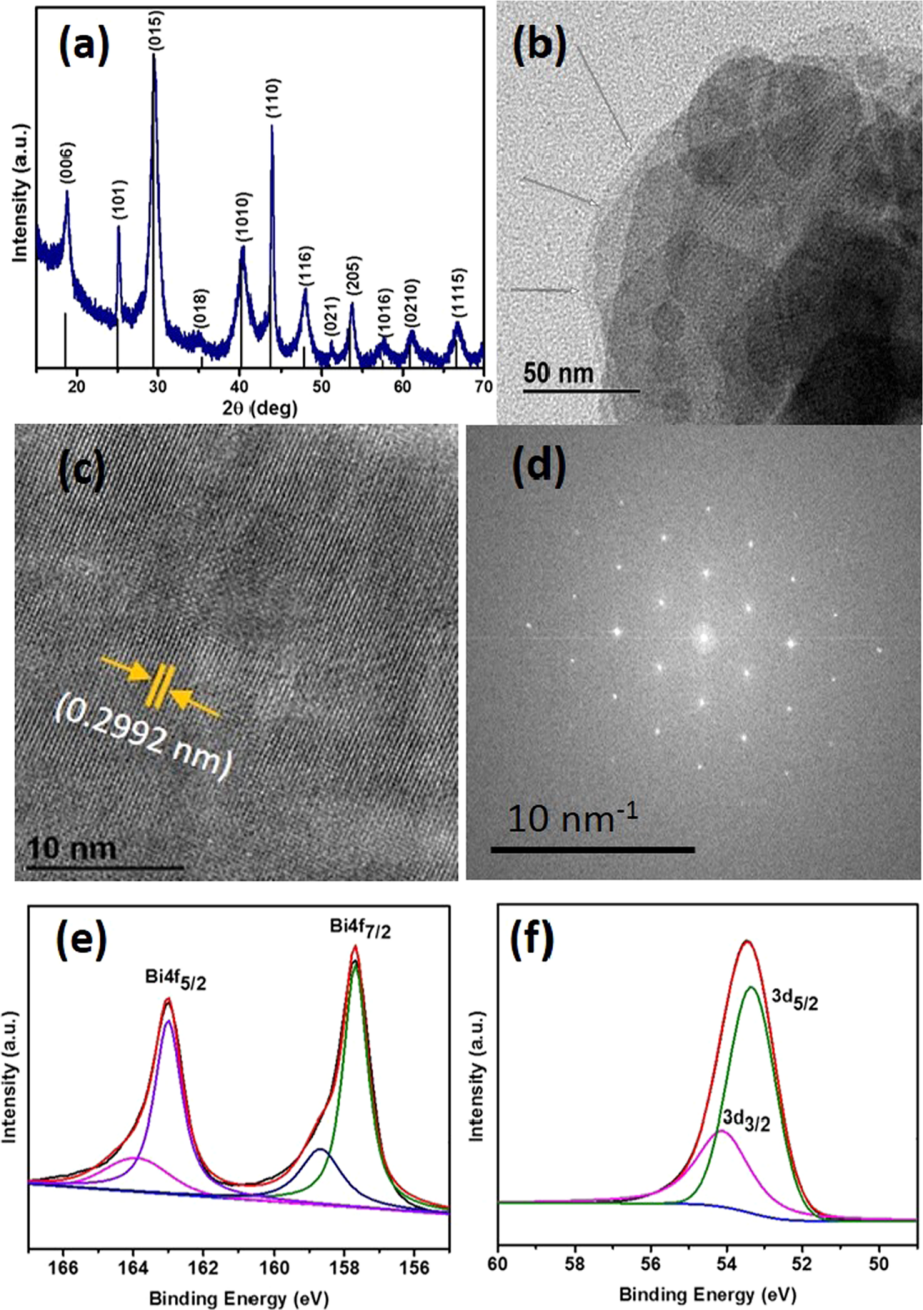
(a) p-XRD of Bi_2_Se_3_ nanosheets synthesized in oleylamine, (b) TEM image of the stacked nanosheets, (c) HRTEM showing lattice fringes, (d) SAED pattern, and (e, f) XPS spectra of Bi 4f and Se 3d, respectively, along with the fitted peaks.

The diffraction pattern of Bi_2_Se_3_ nanosheets is shown in Figure 1a. The pattern observed in the p-XRD of the nanosheets shows a good resemblance with the intensity profile of the standard pattern, with the highest intensity peak along the (015) plane. The intense and slightly broad peaks indicate that the synthesized nanosheets comprise thin sheets. The diffraction pattern matches well with the standard Bi_2_Se_3_ rhombohedral phase (ICDD # 01-089-2008). No peaks belonging to elemental bismuth or selenium were observed.

The nanosheets were relatively thin, as observed by the TEM images (Figure 1b), where overlapped thin layers of Bi_2_Se_3_ can be seen clearly; however, due to stacked sheets, a single separate nanosheet was not observed. The stacked layers of the nanosheets can be seen clearly (indicated by the arrows in Figure 1b), which can be distinguished based on the color complexion of the layers, as in comparison to thickly stacked sheets, mono to few nanosheets seem fairly transparent to the electron beam. The anisotropic nature of the unit cells determines the preferential growth into nanosheets. The basic unit cell of layered Bi_2_Se_3_ is composed of five atomic layers, which are arranged in the order of Se(I)–Bi–Se(II)–Bi–Se(I) sheets.^29^ The superscripts (I) and (II) are used to differentiate between the two Se atoms, which are arranged in different surroundings (Figure S2, SI). The unit cell is described as a quintuple layer (QL) and the covalent character predominates within the QL, whereas two adjacent QLs are linked to each other with weak van der Waals forces, giving rise to a highly anisotropic structure. The weak interactions between the layers allow the generation of mono to a few QLs under judicious reaction conditions. The crystalline nature of these sheets was investigated by HRTEM and SAED analyses. Clear lattice fringes in the HRTEM image (Figure 1c) were observed, and the lattice spacing corresponds to the (015) plane. The crystallinity and high quality of nanosheets were also evident from the well-defined spots as observed in the SAED pattern (Figure 1d). Figure S3 (SI) shows the stacking of thin nanosheets, and due to the thin nature of the sheets, they are flexible enough to buckle and fold to acquire different types of shapes. The nanosheets were of variable sizes and range between tens of nanometers to hundreds of nanometers. The broad peaks in the p-XRD pattern also indicate the presence of very thin nanosheets of Bi_2_Se_3_.

Similarly, the surface composition and chemical states of the synthesized Bi_2_Se_3_ nanosheets were further analyzed by XPS analysis. The survey spectrum of Bi_2_Se_3_ shows the presence of Bi and Se along with carbon, nitrogen, and oxygen (Figure S4, SI). The sample was synthesized in oleylamine; therefore, in this case, the comparatively intense C 1s peak at the binding energy of 284.5 eV indicates that the carbon content may include both adventitious carbon and carbon from the capping agent, i.e., oleylamine, present at the surface of the material. The surface capping by oleylamine is further evidenced by the presence of the N 1s peak at the binding energy of 398.6 eV. Bi_2_Se_3_ nanosheets can be oxidized easily at room temperature, which was evident from the presence of the O 1s peak at a binding energy of 530.2, although due to surface passivation, the extent of oxidation was lower. The Bi 4f spectrum was fitted with Bi 4f_7/2_ and Bi 4f_5/2_ peaks present at binding energies of 157.7 and 163.1 eV, respectively. The minor fitting peaks centered at 158.7 and 164.1 eV may show slight oxidation of the Bi_2_Se_3_ nanosheets (Figure 1e). It is worth noting that the binding energies of Bi 4f peaks in Bi_2_Se_3_ are blue-shifted as compared to elemental bismuth due to charge transfer from Bi to Se.^30^ The Se 3d spectrum can be fitted perfectly by Se 3d_5/2_ and Se 3d_3/2_ peaks present at binding energies of 53.3 and 54.1 eV (Figure 1f), respectively.

The thickness of the Bi_2_Se_3_ nanosheets was determined by AFM analysis (Figure S5, SI). The AFM image indicates the formation of nanosheets in various sizes, and the dimensions of some sheets were in microns. The height profiles at different points indicate a thickness of almost 4 nm. A single layer of Bi_2_Se_3_ is composed of covalently bonded five-atom Se–Bi–Se–Bi–Se chains, and the thickness of a single-layer Bi_2_Se_3_ slab along the [001] direction is 0.96 nm. Furthermore, it has been reported that the determination of the height profile by AFM is exaggerated by almost 0.2–1 nm due to the overlayer of the capping agent used in the synthesis.^31^ Therefore, the observed height profile of 4 nm is nearly equal to 3–4 layers of Bi_2_Se_3_ nanosheets.

**Figure 2:**
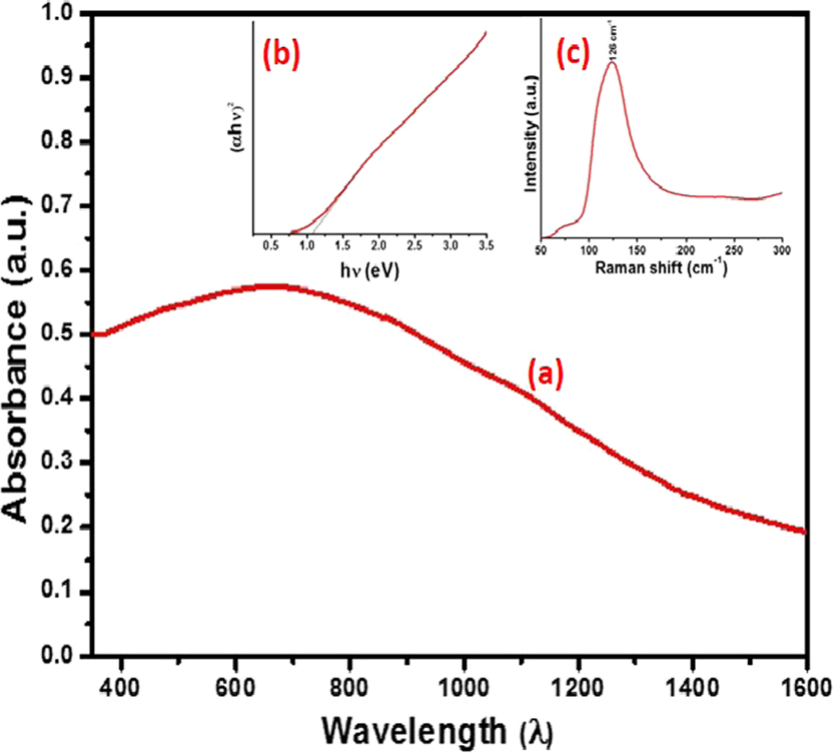
(a) UV–vis–NIR absorption spectrum of Bi_2_Se_3_ nanosheets; the inset shows (b) the band gap by the Tauc plot and (c) Raman shift for Bi_2_Se_3_ nanosheets.

UV–vis–NIR spectroscopy was used to determine the optical properties of the Bi_2_Se_3_ nanosheets. The absorption spectrum of well-dispersed Bi_2_Se_3_ nanosheets (Figure 2a) in acetone resulted in a continuous broadband with λ_max_ at 660 nm. A broad absorption behavior was previously observed for Bi_2_Se_3_ nanosheets, where it was reported that the broadness might be attributed to the local surface plasmon resonance (LSPR).^32^ The energy gap of the Bi_2_Se_3_ nanosheets is estimated to be 1.0 eV (Figure 2b). A shift in the energy gap is probably due to the decreased thickness of the sheets as compared to the parent bulk counterpart. The Raman analysis was performed using a 514 nm laser excitation at 25% power. The characteristic vibration modes for Bi_2_Se_3_ are A_1g_^1^ out-of-plane (72 cm^–1^), E_g_^2^ in-plane (130 cm^–1^), and out-of-plane A_2g_^1^ (174 cm^–1^).^33^ However, due to the broadness of the peak centered at 126 cm^–1^, and the relatively high intensity, the other peaks were amalgamated and only appeared as one prominent peak (Figure 2c). The shift toward a lower value and the increased width of the peak can be attributed to phonon softening and enhanced electron–phonon coupling in a thin-layered structure.^34^ Similarly, for layered materials, the thickness of the nanosheets significantly affects the width, position, and shape of the vibrational mode.^35^

### Bismuth Nanosheets

**Figure 3:**
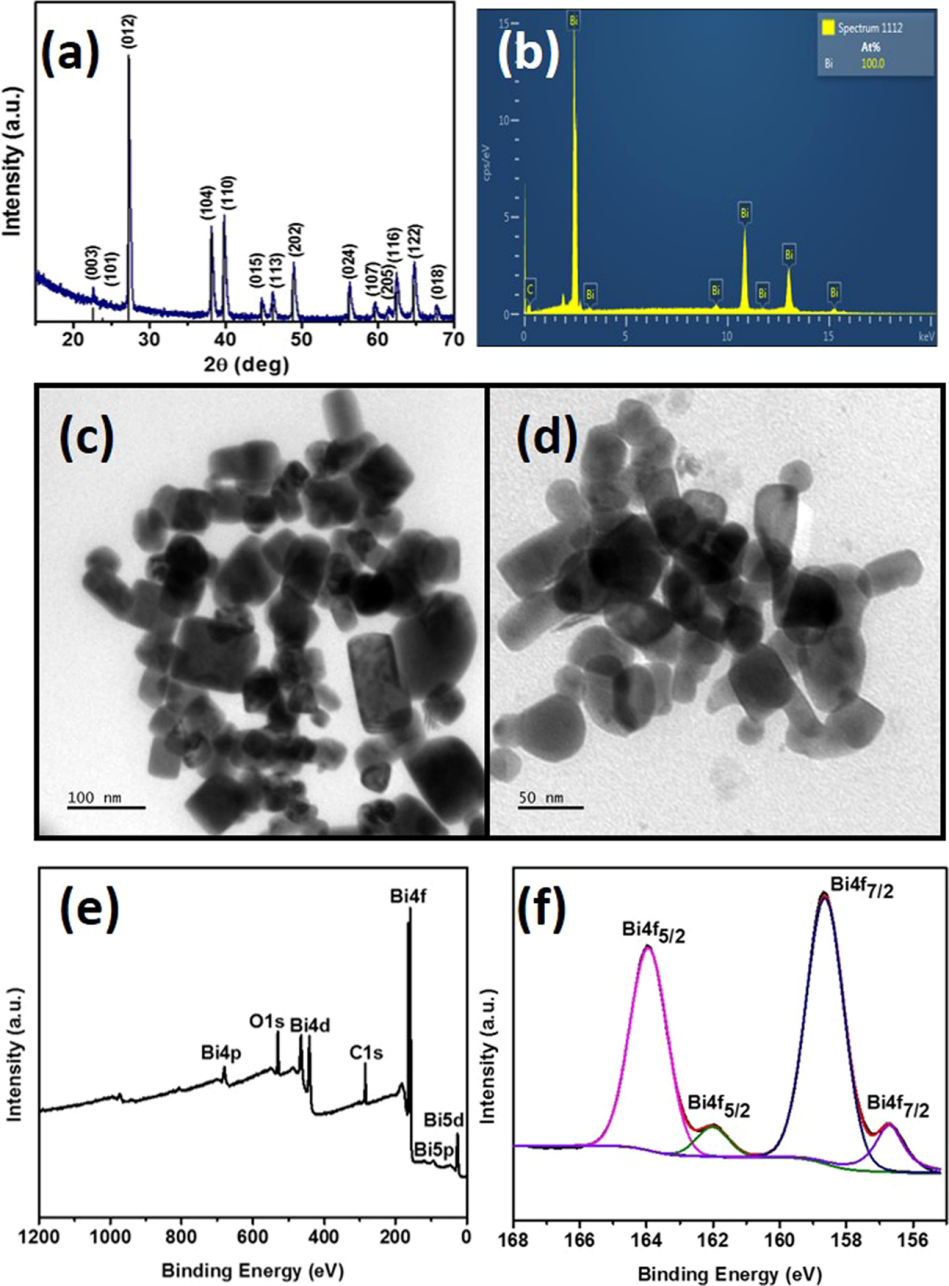
(a) p-XRD analysis of Bi nanosheets, (b) EDX analysis, (c, d) TEM images, (e) survey scan and (f) high-resolution Bi 4f spectrum.

Bismuth was synthesized by the decomposition of the metal–organic precursor in OLA in the presence of TOP. It was noted that the dispersion of the complex in TOP resulted in a change of color of the precursor, which shows the interaction of TOP with the precursor. The dispersed precursor was immediately injected into preheated OLA, and the nature of the residue was observed by p-XRD. The p-XRD analysis indicates the formation of bismuth rather than Bi_2_Se_3_. The peaks were sharp and matched accurately to rhombohedral Bi (ICDD # 01-085-1330) with the *R*3̅*m* space group (Figure 3a). It was interesting to note that, although selenium is directly attached with bismuth in the complex, there was no indication of the formation of Bi_2_Se_3_, even as a minor impurity phase.

The fact that the decomposition of the precursor gave Bi_2_Se_3_ nanosheets when dispersed in ODE shows that the formation of bismuth exclusively is primarily due to the reducing effect of TOP. The interaction of TOP with the complex was investigated by UV–vis analysis. The complex was dissolved in chloroform to monitor the absorption behavior, and a greenish-yellow-colored solution was obtained. A strong absorption was observed below 500 nm for the complex (Figure S6, SI). When a small quantity of TOP was added to the solution, the color of the solution started to change from greenish-yellow to brownish. It shows that the complex begins to decompose in the presence of TOP. The absorption spectrum of the solution after the addition of TOP further confirms the decomposition of the complex as the absorption curve flattens completely.

Later, the complex was dissolved in TOP at room temperature and sonicated for about 15 min. The color of the complex starts to change after ≈5 min to brownish-black. After 15 min of sonication, acetone was added and the decomposition product was washed and separated by centrifugation. The p-XRD analysis of the black powder obtained showed the formation of an amorphous product (Figure S7, SI). It shows that the complex undergoes decomposition even at room temperature, though the resulting product is amorphous. TOP has been used previously as a reducing agent, as trivalent phosphorus in TOP can be easily oxidized from trivalent to the pentavalent state. Lee et al. converted graphene oxide to reduced graphene using TOP, which itself was converted to TOPO during the reduction process.^36^ Mews et al. reported the synthesis of Bi nanoparticles by reducing BiCl_3_ and Bi[N(SiMe_3_)_2_]_3_ precursors using only TOP. ^31^P-NMR spectroscopy confirmed the complete oxidation of TOP to TOPO.^37^ However, there is no report on the reduction of bismuth-based molecular precursors to elemental bismuth.

Molecular precursors have been used previously for selective phase-tuning of semiconducting materials by varying experimental conditions, in both colloidal and solventless routes.^17c,17d^ Here, the metal-to-selenium (i.e., Bi–Se) bond or the selenium-to-carbon (Se–C) bond was selectively dissociated using TOP or ODE to prepare bismuth or Bi_2_Se_3_ nanosheets, respectively. The sample was analyzed by EDX analysis to observe the presence of selenium, which might indicate the formation of Bi_2_Se_3_ as an impurity phase; however, the EDX analysis shows the presence of only bismuth (Figure 3b). The SEM micrographs did not show any definite morphology, and particles with different sizes were randomly oriented (Figure S8, SI). To have a better idea of the morphology, the sample was also analyzed by the TEM analysis, which shows aggregated particles with a mixed morphology (Figure 3c,d). Interestingly, the formation of sheet-like structures was also observed, along with some smaller particles. Sheets were of cubic to rectangular morphology and mostly agglomerated with other particles with an irregular morphology.

Bismuth is easily oxidized in the air; therefore, the surface composition and chemical state of the synthesized Bi were further analyzed by XPS analysis (Figure 3e,f). The survey spectrum of Bi clearly demonstrates the presence of bismuth along with carbon and oxygen (Figure 3e). The C 1s peak at a binding energy of 284.4 was attributed to the presence of adventitious carbon typically found on the surface of materials exposed to air.^38^ Although oleylamine and trioctylphosphine were used as surfactants, their presence on the surface is ruled out by the fact that there is an absence of peaks for binding energies of nitrogen or phosphorus. Since no other peak was detected in the C 1s spectrum, it indicates that there is an absence of any bond between bismuth and carbon. As the surface of bismuth was not well-capped by the surfactants, it became prone to surface oxidation, and the extent of oxidation was relatively greater than Bi_2_Se_3_ nanosheets. Consequently, the O 1s peak at a binding energy of 530.2 is attributed to lattice oxygen, combined with bismuth to form oxide. The relatively low intensity of the O 1s peak, in comparison to pure Bi_2_O_3_, indicates that there is partial surface oxidation and bismuth was not completely converted to its oxide. Besides atmospheric oxygen, the use of solvents, such as methanol and acetone for washing, may also have contributed to the surface oxidation of bismuth. Likewise, to further confirm the chemical state of Bi, a high-resolution spectrum shows the splitting of the main peak for Bi 4f (Figure 3f). The deconvoluted peaks at 156.5 and 162 eV correspond to the bismuth, whereas peaks centered at 158.7 and 164.1 eV, respectively, represent oxidized bismuth due to the surface oxidation of nanosheets, indicating the presence of both elemental bismuth and oxidized bismuth, in agreement with the previously reported literature.^39^ It has also been reported that polishing the surface of bismuth can reduce the oxide layer/content significantly.^40^

**Figure 4:**
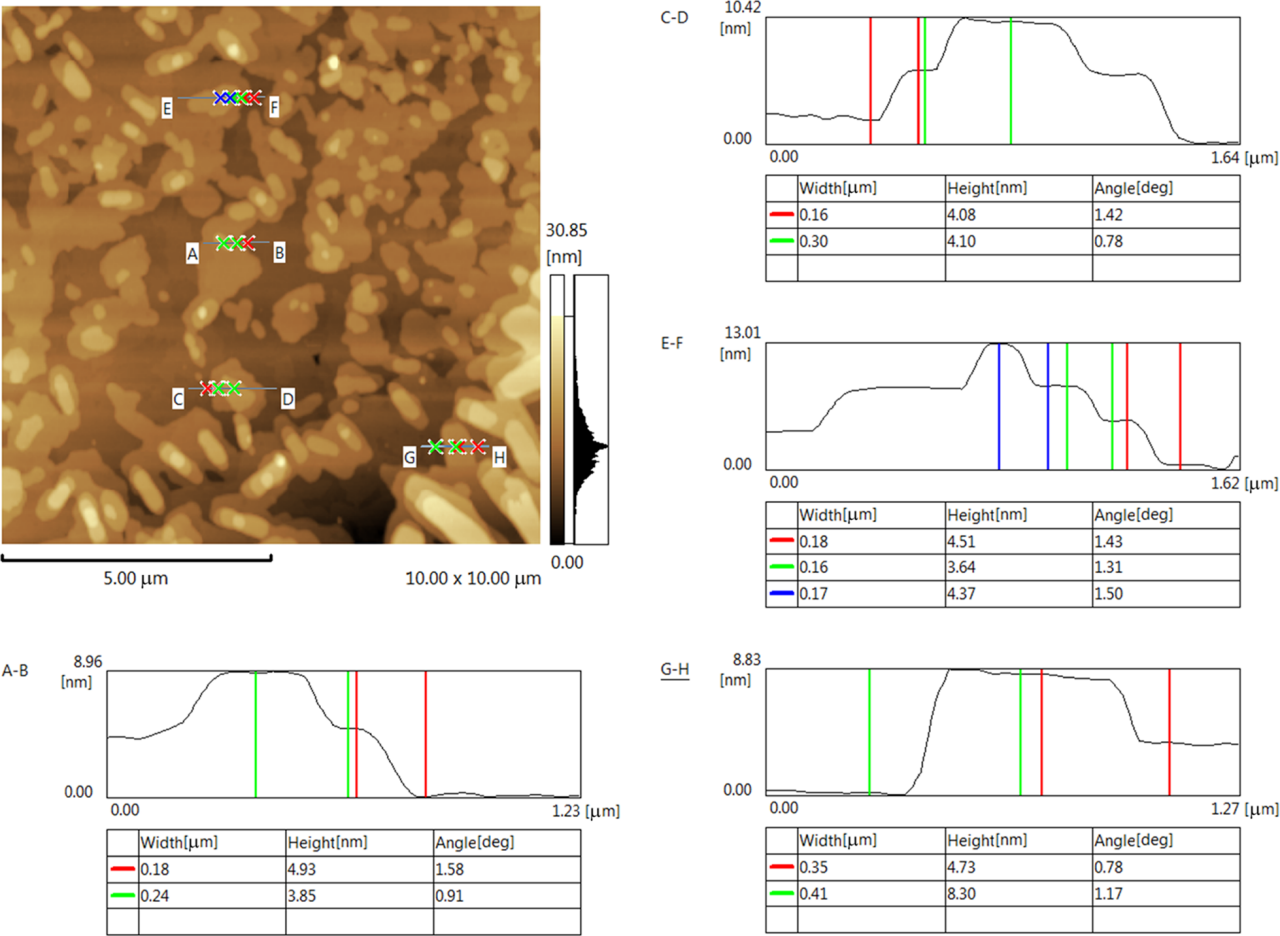
AFM image showing height profiles of the Bi nanosheets at different points.

Due to the agglomeration and irregular morphology, the TEM images were inconclusive in determining the actual morphology of bismuth nanoparticles, i.e., whether they are nanosheets or have a particle-like morphology. Therefore, AFM was used to further investigate the morphology of bismuth. As shown in Figure 4, the AFM image also confirms the presence of agglomerated particles with highly diverse shapes and sizes. The sizes of the particles vary between less than 100 nm to microns. Due to agglomeration, the height profiles at different points also showed variation, with the lowest thickness of 3.6 nm to the highest thickness of 8.3 nm. The theoretical monolayer thickness of bismuth is 0.395 nm,^39a^ which shows that the sheets with the lowest thickness are composed of 8–9 layers of bismuth. The stacking of smaller sheets over larger sheets results in increased height profiles, as shown in Figure 4.

The harsh conditions (i.e., use of oleylamine and high temperature) may have prevented the formation of sheets with uniform size distribution due to their breakdown. A similar observation was reported by Pumera et al., where the exfoliation of bulk bismuth in different organic solvents resulted in the formation of particles with diverse shapes and sizes.^41^ The probable reason might be that though the layered structure of bismuth is similar to graphene sheets in graphite, the interaction between layers is stronger, which causes a close competition between exfoliation and downsizing. It is anticipated that employing softer conditions/ligands may yield bismuth nanosheets with a narrow size distribution and thinner dimensions.

### Bi_2_Se_3_ Thin Films

Since colloidal synthesis provided different products by changing the reaction parameters, the rationale behind investigating the precursor’s decomposition by the vapor deposition method is to examine the effect of the synthetic route on the final product. The precursor can behave differently under different synthetic conditions, and since chalcogenides have high partial pressure and bismuth can be reduced under mild reaction conditions, it is worth investigating whether bismuthene films can be deposited from the same precursor at elevated temperatures.

The deposition was carried out on the glass substrates by AACVD. The complex was readily soluble in common organic solvents, such as CHCl_3_, THF, and toluene. It was observed that the complex starts to decompose in THF after a while during the generation of an aerosol, and toluene aerosol requires a comparatively longer duration to finish; hence, chloroform was used as a solvent for deposition. As observed by the TGA analysis, the complex decomposes completely around 385 °C; therefore, a deposition temperature between 400 and 500 °C was used. A light deposition was observed on the slides placed at the start of the hot zone, but a thicker deposition on the slides placed at the center to the end of the glass tube was detected. All films were black, compact, and showed moderate adherence (can be scratched easily by a spatula). A closer examination of the film also suggested the presence of some particulate crystallites at the film surface. It may indicate that the decomposition of the precursor did not entirely occur on the heated substrates, rather slightly above the surface of the substrates—a phenomenon also referred to as snowing of the films—resulting in poorly adherent films with a particulate nature. All deposition experiments were carried out until complete evaporation of the solution, which took almost 30 min.

The diffraction patterns of the films deposited at different temperatures are shown in Figure S9, SI. At 400 °C, only an intense peak at 2θ ≈ 31.1° was observed, which matches with the Bi_2_Se_3_ phase (ICDD # 01-085-0519). The films are highly crystalline and textured along the (221) plane, as indicated by the single intense peak detected in this plane. At 450 °C, other peaks were also observed, but they appeared only as minor peaks due to their low relative intensity as compared to the high-intensity peak along the (221) plane. At 500 °C, the intensity of this peak further increases and no other peaks were observed, probably masked by the intense peak along the (221) plane. The broad hump observed in all cases is due to the amorphous glass substrate. The observations in p-XRD patterns show that the deposition temperature may have only little effect on the growth of the crystallite size and morphology, as the preferred orientation of the crystallites in thin films deposited at different temperature remains the same.

The effect of temperature on size, morphology, and stoichiometry was analyzed by SEM analysis. At a deposition temperature of 400 °C, the formation of granular particles, which were uniformly distributed on the glass substrate, was observed (Figure S10a,b, SI). The particles had a broad size distribution. The films were slightly Se-enriched, and EDX analysis indicates an average Se/Bi ratio of 1.73, which is somewhat higher than the required stoichiometry of 1.50 for Bi_2_Se_3_ (Figure S11a, SI). The homogeneity of the deposited films was investigated by elemental mapping, which shows a uniform distribution of bismuth and selenium over the substrate (Figure S12a–c, SI).

The growth at a higher temperature of 450 °C indicates only a slight alteration in the size of crystallites. A small increase in the size of crystallites can be observed, but generally, the films showed a similar morphology (Figure S10c,d, SI). The stoichiometry of the films also changed, and an average Se/Bi ratio of 1.65 was obtained from the EDX analysis. The films are still selenium-rich but to a lesser extent as compared to the films deposited at a temperature of 400 °C (Figure S11b, SI). The elements were homogeneously distributed, as indicated by the elemental mapping (Figure S12d–f, SI).

Further increase in the deposition temperature to 500 °C led to the deposition of layered or sheetlike structures (Figure S10e,f, SI), which appear to form clusters. The structure of Bi_2_Se_3_ consists of layers that are interconnected by weak van der Waal’s forces. The high temperature ruptures the delicate forces and results in splitting the three-dimensional structure into clusters of a few layers. The stoichiometry was also significantly affected, as the films deposited were deficient in selenium with an average Se/Bi ratio of 1.37, as shown by EDX (Figure S11c, SI). The loss of chalcogens at higher temperatures is a well-known observation due to their high partial pressure, and this trend is clearly shown in the stoichiometry of all deposited films deposited at different temperatures. The homogenous distribution of Bi and Se in films was indicated by EDX analysis (Figure S12g–i, SI).

### Electrochemical Performance of Bi_2_Se_3_

**Figure 5:**
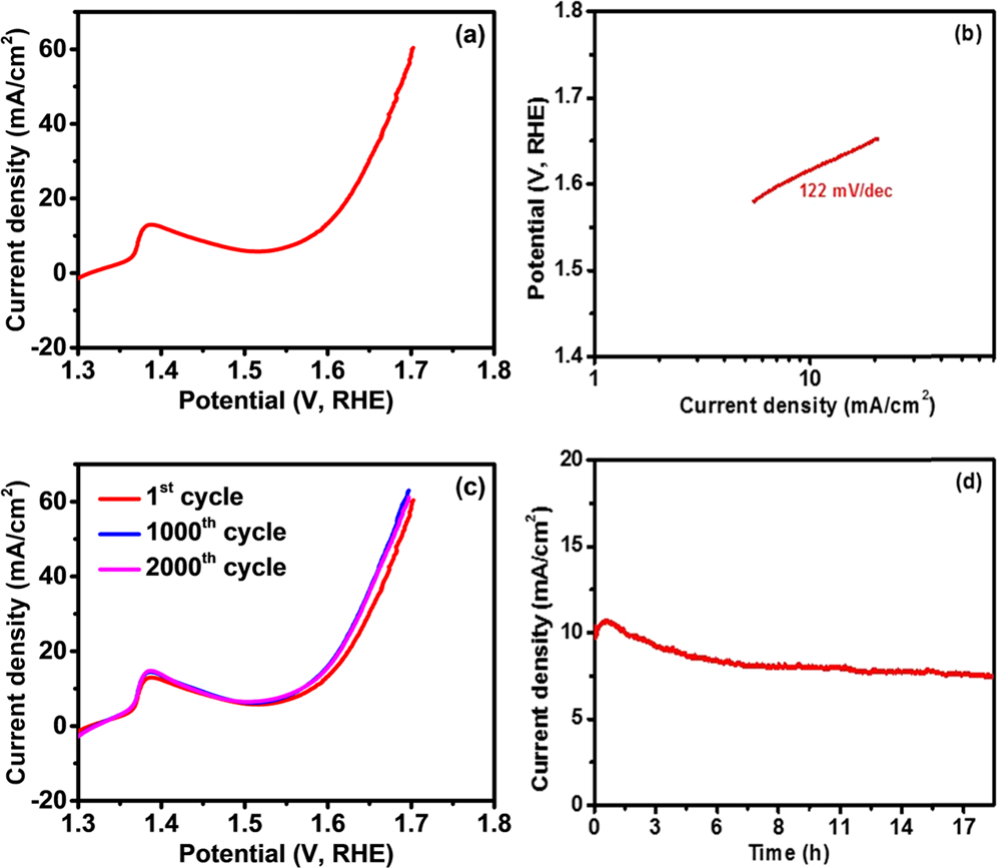
(a) Polarization curve, (b) corresponding Tafel slope, (c) cyclic stability, and (d) chronoamperometry measurements of the Bi_2_Se_3_ electrode for OER.

The detail regarding the experimental setup is provided in the Supporting Information. A potential application of the Bi_2_Se_3_ nanosheets, synthesized by the solvothermal method, was tested for both hydrogen and oxygen evolution in an alkaline medium. Figure 5a shows a polarization curve for the Bi_2_Se_3_ electrode. The Bi_2_Se_3_ electrode displayed an overpotential of 385 mV at 10 mA cm^–2^. A blank test of the substrate without Bi_2_Se_3_ was performed to supplement that the electrocatalytic performance originates from the active Bi_2_Se_3_ rather than the substrate (Figure S13a, SI). It was observed that, for Ni foam to obtain a current density of 10 mA cm^–2^, an overpotential of 440 mV is required, which clearly indicates that the catalytic activity is coming from Bi_2_Se_3_ nanosheets. The observed overpotential was compared with other commonly used oxides or chalcogenide-based nonprecious OER catalysts, suggesting that the synthesized catalyst is better than or comparable to the electrocatalysts prepared by different methods (Table S1). To determine the OER kinetics of the Bi_2_Se_3_ catalyst, the Tafel slope was calculated by plotting the potential versus the log of current density. As seen in Figure 5b, the Tafel slope for the Bi_2_Se_3_ electrode was calculated to be 122 mV dec^–1^.

The electrochemical stability of the Bi_2_Se_3_ electrode was studied using cyclic voltammetry and chronoamperometry. Figure 5c indicates the polarization curves of the Bi_2_Se_3_ electrode at the 1st, 1000th, and 2000th cycles. As evident from the polarization curves at various cycles, the Bi_2_Se_3_ electrode is electrochemically stable up to 2000 cycles. The electrochemical stability was further tested using chronoamperometry. As shown in Figure 5d, the current density of the Bi_2_Se_3_ electrode was constant for almost 18 h of study.

**Figure 6:**
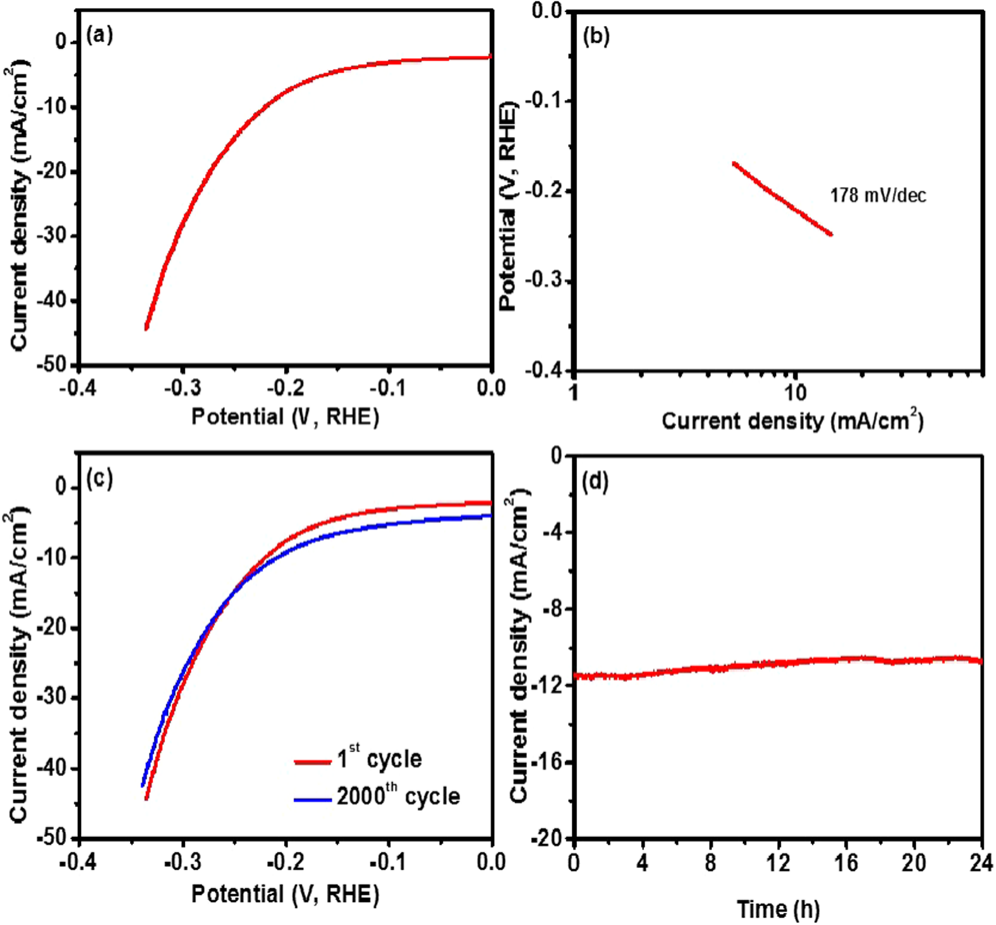
(a) Polarization curve, (b) corresponding Tafel slope, (c) cyclic stability, and (d) chronoamperometry measurements of the Bi_2_Se_3_ electrode for HER.

Bifunctionality of the Bi_2_Se_3_ electrode as an electrocatalyst for the HER process was also tested in an alkaline medium. Figure 6a shows the polarization curve of the Bi_2_Se_3_ electrode. The Bi_2_Se_3_ electrode required an overpotential of 220 mV to achieve a current density of 10 mA cm^–2^ with a Tafel slope of 178 mV dec^–1^ (Figure 6b). The Ni foam showed an overpotential of 319 mV at 10 mA cm^–2^, suggesting that catalytic activities are originating from the Bi_2_Se_3_ (Figure S13b, SI). Luo et al. reported that pristine Bi_2_Se_3_ nanosheets required an overpotential of 508 mV to attain a current density of 10 mA cm^–2^, whereas Au-decorated Bi_2_Se_3_ nanosheets needed an overpotential of 380 mV to achieve the same current density.^42^ Yang et al. reported a current density of 0.8 mA cm^–2^ for pure Bi_2_Se_3_ at 300 mV, which can be significantly enhanced by coupling it with MoSe_2_ nanosheets.^43^ Pumera et al. prepared Bi_2_Se_3_ nanosheets by electrochemical exfoliation; however, the exfoliated Bi_2_Se_3_ nanosheets were catalytically inactive toward HER and OER.^44^ It has been proposed that the high activity of Bi_2_Se_3_ is a result of surface oxidation of Bi_2_Se_3_ nanosheets.^45^ The oxidized layer forms a heterojunction and results in the readjustment of the band structure and provides better charge transfer. The oxidized surface could also provide more active edge sites for high HER performance.^43^ Thus, the synergistic effect of surface oxidation is responsible for the better performance of the Bi_2_Se_3_ nanosheets. A comparison of the HER activity of Bi_2_Se_3_ nanosheets with other nonprecious electrocatalysts is shown in Table S2. The durability of the Bi_2_Se_3_ electrode as an HER catalyst was also studied using cyclic voltammetry (Figure 6c) and chronoamperometry (Figure 6d). As seen from both studies, the Bi_2_Se_3_ electrode indicated a highly stable performance up to 2000 cycles of study and over 24 h of chronoamperometric study. Our results suggest that the Bi_2_Se_3_ electrode is highly durable and efficient for both OER and HER processes.

**Figure 7:**
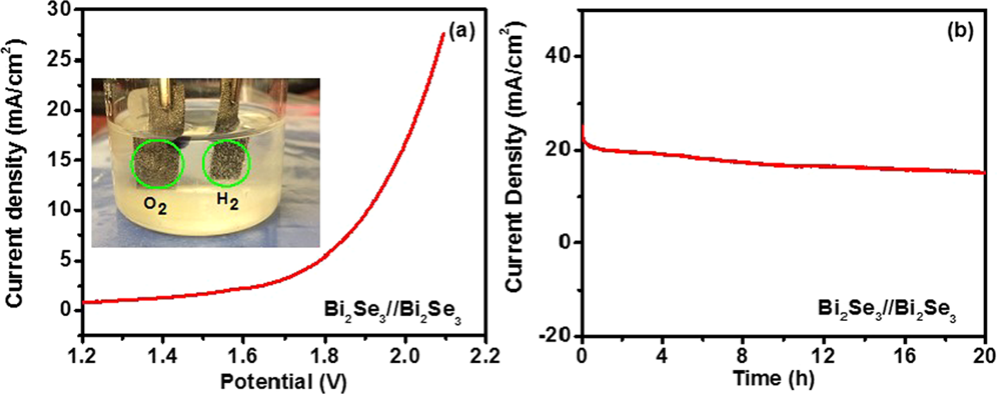
(a) Polarization and (b) chronoamperometry plots for overall water splitting of a two-electrode electrolyzer using a Bi_2_Se_3_/Bi_2_Se_3_ electrocatalyst couple. The inset in (a) displays an optical image of the Bi_2_Se_3_/Bi_2_Se_3_ electrocatalyst showing generated O_2_ and H_2_.

An electrolyzer was fabricated using two Bi_2_Se_3_ electrodes as the anode and cathode for overall water-splitting studies. The fabricated electrolyzer required a cell voltage of 1.9 V to attain a current density of 10 mA cm^–2^ (Figure 7a). The performance of the electrolyzer was further tested using chronoamperometry (Figure 7b). As seen in the chronoamperometry plot, the Bi_2_Se_3_-based electrolyzer displayed considerable electrochemical stability for about 20 h.

### Electrochemical Performance of Bi

**Figure 8:**
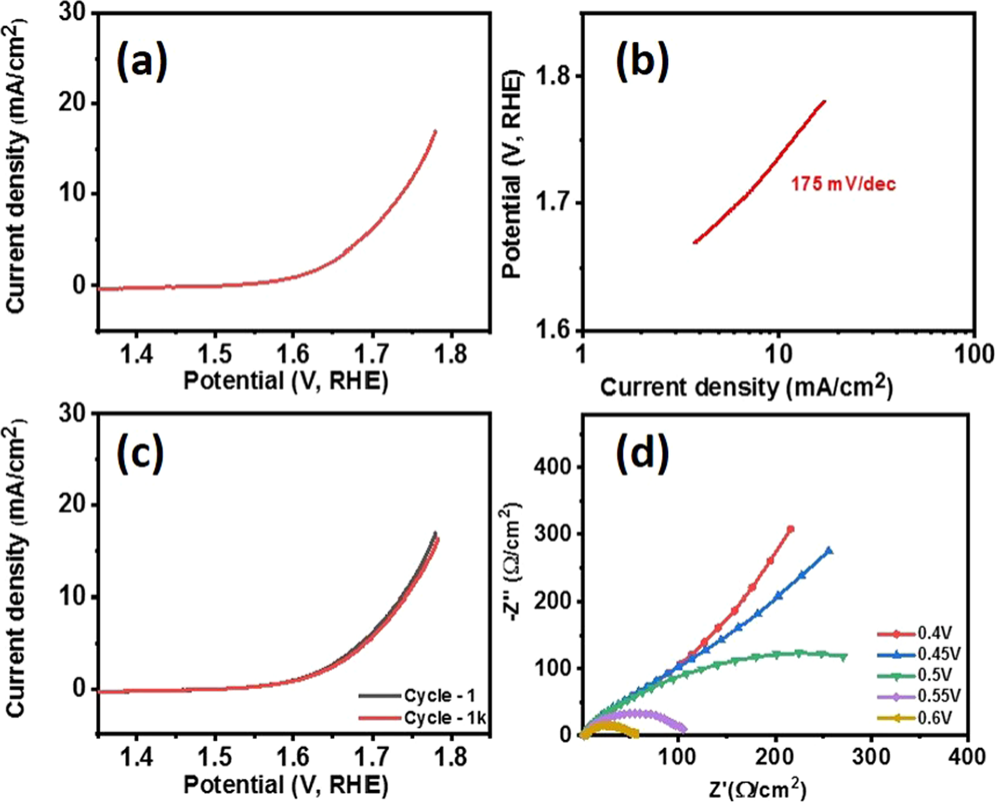
(a) Polarization curve, (b) Tafel slope, (c) stability test, and (d) EIS plots at various potentials for the OER activity of bismuth nanosheets.

Electrocatalytic performance of bismuth nanosheets was also tested for both hydrogen and oxygen evolution in an alkaline medium. Figure 8a shows a polarization curve for the OER activity of the Bi electrode. The Bi electrode displayed an overpotential of 506 mV at 10 mA cm^–2^. It indicates that Bi_2_Se_3_ nanosheets have better OER activity as compared to bismuth nanosheets. The observed overpotential was also compared with other commonly used oxide or chalcogenide-based nonprecious OER catalysts to compare with other electrocatalysts prepared by different methods (Table S1). Likewise, the Tafel slope was calculated by plotting potential versus the log of current density to determine the OER kinetics of the Bi nanosheets. The Tafel slope for the Bi electrode was estimated to be 175 mV dec^–1^ (Figure 8b). The electrochemical stability of the Bi electrode was studied using cyclic voltammetry. Figure 8c indicates the polarization curves of the Bi electrode at 1st and after 1000 cycles. As evident from the polarization curves, the Bi electrode is electrochemically stable up to 1000 cycles. Electrochemical impedance spectroscopy (EIS) was performed to determine the overall series resistance of the sample (Figure 8d). It is well-known that the smaller the radius of the semicircle, the lower will be the resistance. The graph indicates an inverse relation between the arc radius of each sample and the applied voltage. The significant decrease in radius with the increase in applied voltage suggests that, at higher voltage, bismuth leads to more effective charge separation and faster interfacial charge transfer.

**Figure 9:**
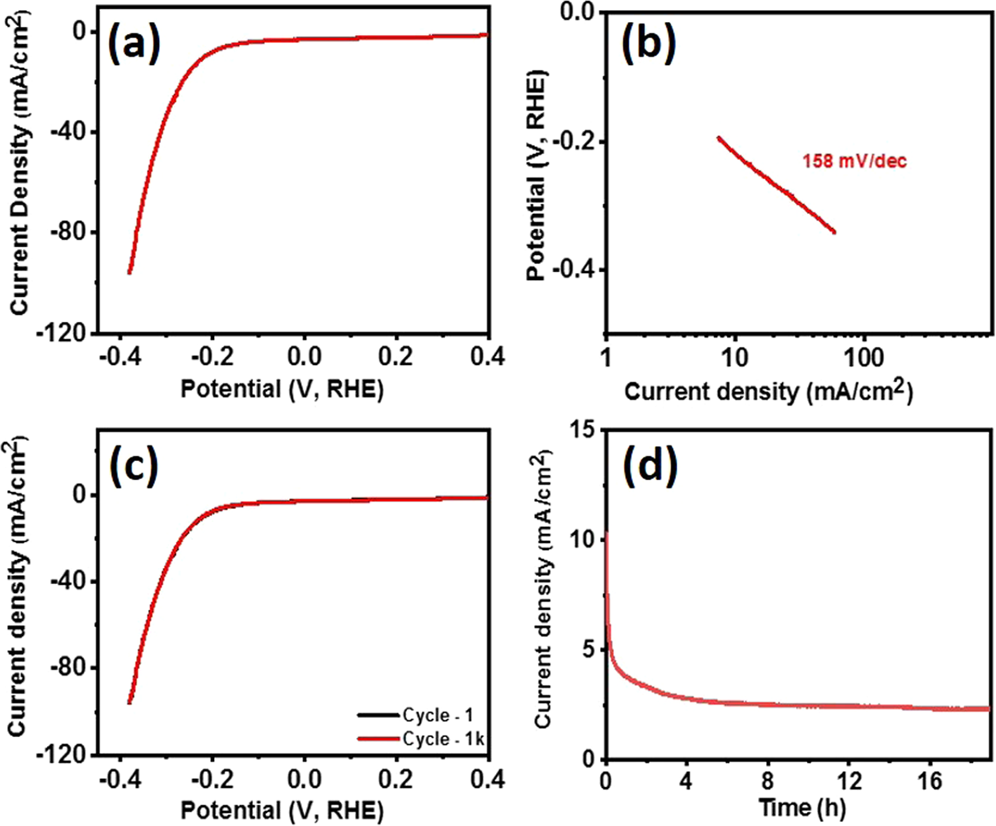
(a) Polarization curve, (b) Tafel slope, (c) stability test, and (d) chronoampere test for bismuth nanosheets.

The electrocatalytic performance of the Bi electrode for the HER process was also tested in an alkaline medium. Figure 9a shows the polarization curve of the Bi electrode. The bismuth electrode required an overpotential of 214 mV to achieve a current density of 10 mA cm^–2^ with a Tafel slope of 158 mV dec^–1^ (Figure 9b). The Ni foam showed an overpotential of 319 mV at 10 mA cm^–2^, suggesting that catalytic activities are originating from the Bi (Figure S7b, SI). A comparison of the HER activity of Bi with other nonprecious electrocatalysts is shown in Table S2. The durability of the bismuth electrode as an HER catalyst was also studied using cyclic voltammetry (Figure 9c) and chronoamperometry (Figure 9d). As seen from both studies, the Bi_2_Se_3_ electrode indicated a stable performance up to 1000 cycles of study, and after an initial decrease in the current density, which may be probably due to the surface oxidation of bismuth, a steady state was indicated for over 18 h of chronoamperometric study.

### Photoelectrochemical Water Reduction

**Figure 10:**
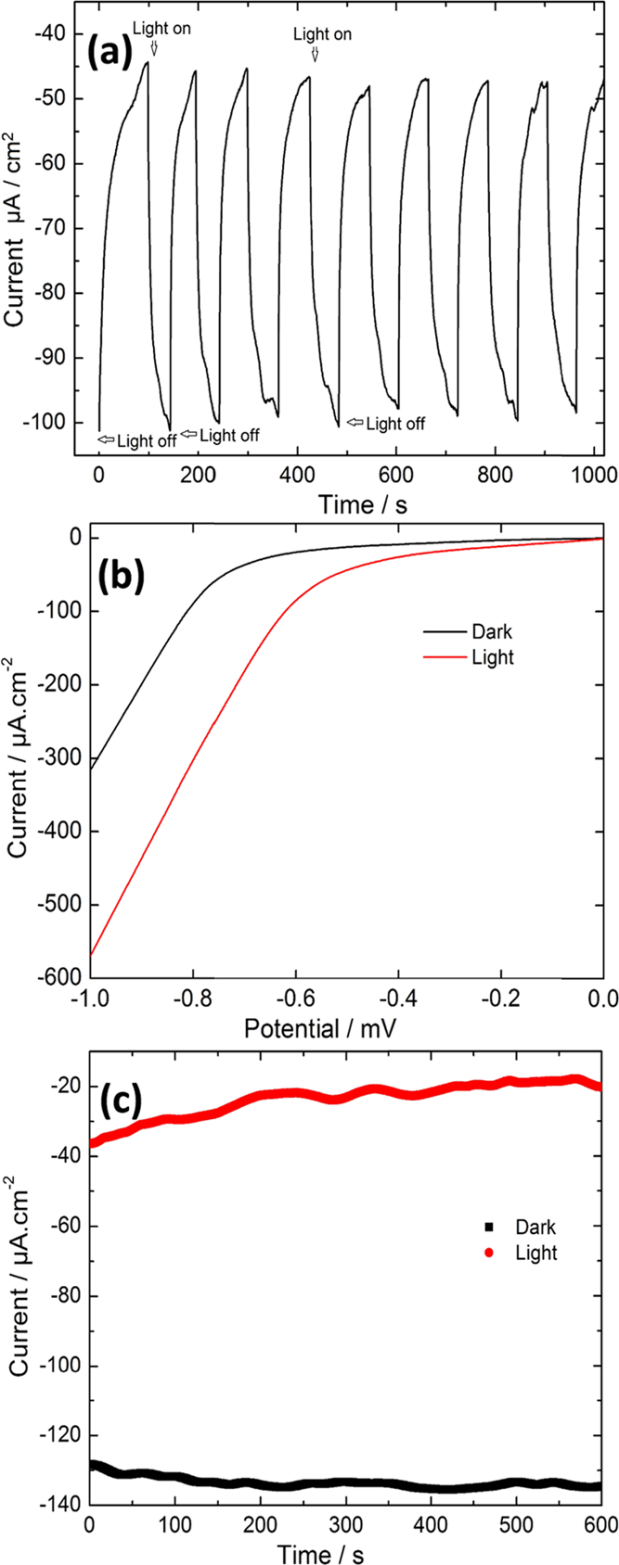
(a) Chronoamperometric measurements with the Bi_2_Se_3_/FTO electrode at OCP using chopping 1 sun simulated illumination, (b) LSV curves (vs SHE) with the Bi_2_Se_3_/FTO electrode in dark and under continuous 1 sun simulated illumination, and (c) chronoamperometric stability measurements with Bi_2_Se_3_/FTO at OCP in dark and under continuous 1 sun simulated illumination.

Colloidally synthesized Bi_2_Se_3_ nanosheets deposited on FTO-glass substrates were used for photoelectrochemical studies. The complete detail regarding electrode preparation and electrochemical setup for photoelectrochemical investigation is shown in the Supporting Information. Bi_2_Se_3_ nanosheets were observed to exhibit cathodic photocurrent. The chronoamperometric performances of the Bi_2_Se_3_/FTO photocathodes, obtained at open-circuit potential (OCP) as a function of time, under simulated sunlight illumination are shown in Figure 10a. Once the current response was stable, the light was cut off at regular intervals. It was evident that a significant cathodic current was generated for H_2_ generation only when the light was illuminated at the surface of the Bi_2_Se_3_/FTO electrode. When the light was cut off, the photocurrent density instantaneously became negligible. This infers that the current generated was only due to the illuminated light and not attributed to any intrinsic properties of the Bi_2_Se_3_ nanostructures.^46^ The photocathodic current generated due to H_2_ evolution for the Bi_2_Se_3_/FTO electrode was in the range of −48.5 to −56.3 μA cm^–2^.

The LSV curves obtained with the Bi_2_Se_3_/FTO electrode are displayed in Figure 10b. The enhancement in the cathodic current density was observed for the Bi_2_Se_3_/FTO electrode over the entire potential range tested. The change in current density in the dark was from −28.0 μA cm^–2^ at 0 V to −87.7 μA cm^–2^ at 0.6 V, while under simulated solar light, the current density was −109.8 μA cm^–2^ at 0 V and reached up to −300.5 μA cm^–2^ at 0.6 V.

The Bi_2_Se_3_/FTO photocathode stability was also assessed under dark and sunlight illumination, as displayed in Figure 10c. The electrode showed a very stable response over 600 s of testing time. The dark current was very stable, while a noisy photocurrent was observed due to H_2_ evolution and accumulation of H_2_ bubbles at the surfaces in the cases of both of the electrodes.^47^ A small decrease in photocurrent response was observed over time, which could be recovered once the solution was stirred to remove the H_2_ bubbles from the electrode surface. Thus, the Bi_2_Se_3_/FTO photocathode acted as an efficient water reduction catalyst in the neutral sodium sulfate solution and could be a promising candidate for cathodic water-splitting applications.

## Conclusions

A facile synthetic route was used to prepare a mono-selenobenzoate complex of bismuth (tris(selenobenzoato)bismuth(III)) at room temperature. The synthesized molecular precursor was used for the preparation of Bi or Bi_2_Se_3_ nanosheets by the colloidal method. The reactions were performed at relatively mild reaction conditions. The precursor decomposes at room temperature in the presence of oleylamine or in TOP, yielding amorphous Bi_2_Se_3_ or Bi, respectively. Therefore, a range of temperatures can be used to prepare the desired product. It was observed that TOP acted as a reducing agent, and the decomposition of the complex in the presence of TOP was studied by UV–vis spectroscopy and p-XRD. When 1-ODE or oleylamine was used as a dispersion medium, Bi_2_Se_3_ nanosheets were formed exclusively. Oleylamine has a dual role, i.e., it acts as a capping agent and initiates the degradation of the complex. XPS analysis indicated that the handling of the samples in normal atmospheric conditions and the use of solvents such as methanol and acetone are probably responsible for the surface oxidation of both materials. The height profiles by AFM suggested that Bi_2_Se_3_ nanosheets were composed of almost 3–4 layers, whereas Bi nanosheets were composed of 9–10 layers. Likewise, the decomposition of the precursor in the vapor phase resulted in the formation of Bi_2_Se_3_ films. The stoichiometry of the thin films, as determined by EDX, varies significantly (from selenium-rich to selenium-deficient) with a change in temperature. The crystallites’ size also increases with an increase in temperature; however, the films showed poor adherence to the substrate and nonuniformity.

Bi_2_Se_3_ nanosheets showed high activity for electro- and photoelectrochemical water splitting. The OER and HER catalytic performances indicate overpotentials of 385 mV at 10 mA cm^–2^ and 220 mV, with Tafel slopes of 122 and 178 mV dec^–1^, respectively. The electrodes indicated a highly stable performance up to 2000 cycles of study and over 24 h of chronoamperometric study. In comparison, Bi showed a much lower OER activity, requiring an overpotential of 506 mV to achieve a current density of 10 mA cm^–2^ with a Tafel slope of 175 mV dec^–1^. However, the HER performance was slightly better than Bi_2_Se_3_ nanosheets, indicating an overpotential of 214 mV at 10 mA cm^–2^ with a Tafel slope of 158 mV dec^–1^.

Similarly, Bi_2_Se_3_ nanosheets also showed significant photoelectrochemical catalytic properties for water splitting, indicating the material’s high potential as a cheap source for the production of hydrogen from water.

## Supporting Information Available

Electrochemical water splitting; photoelectrocatalytic (PEC) water splitting; stepwise illustration of the proposed decomposition pathway of the tris(selenobenzoato)bismuth(III) complex; Thermogravimetric analysis; TEM images showing stacking, buckling, and folding of the Bi_2_Se_3_ nanosheets; Survey scan of bismuth selenide; AFM image showing height profiles of the Bi_2_Se_3_ nanosheets at different points; UV spectra of the complex, before and after addition of TOP; p-XRD pattern of Bi prepared by decomposition of precursor at room temperature; SEM images of bismuth at different magnifications; p-XRD, SEM, EDX and mapping of Bi_2_Se_3_ thin fims; OER and HER polarization curves for Ni foam; comparison of OER performance of Bi_2_Se_3_ nanosheets with previously reported nonprecious electrocatalysts (PDF)
