# Supplementary material for: Selective Synthesis of Bismuth or Bismuth Selenide Nanosheets from a Metal Organic Precursor: Investigation of their Catalytic Performance for Water Splitting
Source: Inorg Chem. 2021 Jan 19;60(3):1449–61. doi: 10.1021/acs.inorgchem.0c02668 (PMC8716079; doi:10.1021/acs.inorgchem.0c02668)
Supplement: Supplementary file 1 — ic0c02668_si_001.pdf [file ic0c02668_si_001.pdf]

Supplementary Data

Selective Synthesis of Bismuth or Bismuth Selenide Nanosheets from a Metal Organic Precursor: Investigation of their Catalytic Performance for Water Splitting

Shumaila Razzaque,[a] Malik Dilshad Khan,[b,c]* Muhammad Aamir,[d] Manzar Sohail,[e] Sanket Bhoyate,[f] Ram K. Gupta,[f] Muhammad Sher,[g] Javeed Akhtar,[d] and Neerish Revaprasadu [c]*

[a]Key Laboratory of Material Chemistry for Energy Conversion and Storage, Ministry of Education, School of Chemistry and Chemical Engineering, Huazhong University of Science and Technology, Luoyu Road No. 1037, Wuhan, China.

[b]Institute of Physical Chemistry, Polish Academy of Sciences, Kasprzaka 44/52, 01-224 Warsaw, Poland.

[c]Department of Chemistry, University of Zululand, Private bag X1001, Kwa-Dlangezwa 3880, South Africa.

[d]Department of Chemistry, Materials Laboratory, Mirpur University of Science & Technology (MUST), Mirpur-10250 (AJK), Pakistan.

[e]Department of Chemistry, School of Natural Sciences, National University of Science and Technology, H-12, Islamabad 46000, Pakistan.

[f]Department of Chemistry, Pittsburg State University, Pittsburg, KS 66762, USA.

[g]Department of Chemistry, Allama Iqbal Open University, Islamabad, Pakistan.

*Email: malikdilshad@hotmail.com, RevaprasaduN@unizulu.ac.za

Electrochemical water splitting

For electrochemical measurements, versastat 4-500 electrochemical workstation was used for electrochemical measurements (*i.e.* water splitting and supercapacitance). A three-electrode system was used to study the electrocatalytic activities of the synthesized material. A graphite rod and a saturated calomel electrode (SCE) were used as counter electrode and reference electrode respectively, whereas the synthesized material (Bi_2Se_3 nanosheets prepared by colloidal route) on nickel foam was used as a working electrode in 1M KOH electrolyte. For the preparation of a working electrode, first, nickel foam was cleaned using 3 M HCl solution, followed by a cleaning using water and acetone. A paste consisting of the synthesized sample (80 wt.%), acetylene black (10 wt.%) and polyvinylidene difluoride (PVDF, 10 wt.%) was prepared using N-methyl pyrrolidinone (NMP) as a solvent. This paste was then applied to a pre-cleaned and weighted nickel foam. The paste was then dried under vacuum at 60 °C for 10 hours. The loading mass was 1.51 mg/cm^2. The energy storage properties of the electrode were analyzed under similar conditions except for the electrolyte being 3M KOH and the counter electrode was platinum wire. Polarization stability for OER and HER was performed using linear sweep voltammetry (LSV) at various cycles. The LSV curves of OER and HER were reported after iR correction. The resistance for the iR corrections were measured using Electrochemical Impedance Spectroscopy. Supercapacitor studies included galvanostatic charge-discharge (CD) and cyclic voltammetry (CV) measurements. LSV measurements were performed at a scan rate of 1 mV/s, both for oxygen and hydrogen evolution reactions. The potential was converted to RHE using the Nernst equation. Two Bi_2Se_3 electrodes separated by an ion transporting layer were used to fabricate supercapacitor device. The electrodes and ion-transporting layer was soaked in 3M KOH before assembling the device. CV and CD were performed on the device at various scan rates and current densities, respectively.

Photoelectrocatalytic (PEC) water splitting

PEC studies were carried out using fluorine doped tin oxide (FTO) conducting glass substrates. FTO substrates were ultra- sonicated, first in acetone and then in deionized water for 10 minutes each. A slurry of Bi_2Se_3 nanosheets was prepared by dispersing the nanosheets in *iso*-propanol and sonicated for 10 minutes. Bi_2Se_3/FTO electrode was prepared by drop casting 50

μL of the Bi_2Se_3 slurry over 1 cm^2 of the FTO glass substrate, which was then annealed at 80 °C for two hours to harden the substrate layer. An increment of 10 μL of 1% nafion solution was then drop casted over the Bi_2Se_3 layer to make it withstand during the PEC measurements. Once dried Bi_2Se_3/FTO electrode was used for all subsequent PEC studies.

A conventional three electrode cell was used for PEC studies with Bi_2Se_3/FTO electrode as the working electrode, Ag/AgCl chloride (Saturated KCl) as reference electrode and platinum wire as an auxiliary electrode. The linear scan voltammetry (LSV) results are reported against the standard hydrogen electrode (SHE) by adding 0.197 V in all the measured results.[1] All three electrodes were placed in a 5.0 mL quartz cell containing 0.5 M sodium sulfate solution (pH 6.8) as an electrolyte. To remove any dissolved oxygen the cell solution was purged with nitrogen gas for 10 minutes prior to the PEC measurements. A solar simulator equipped with a 150 W Xenon arc lamp (Model 16S-150, Solar Light Company Inc., Glenside PA 19038, USA) was used with a 1 sun light intensity. A mini μ-Autolab potentiostat, PGSTAT101 (MetrohAutolab.B.V.Kanaalweg, 29-G, Utrecht, The Netherlands) with NOVA 2.1 software was employed in all electrochemical experiments.

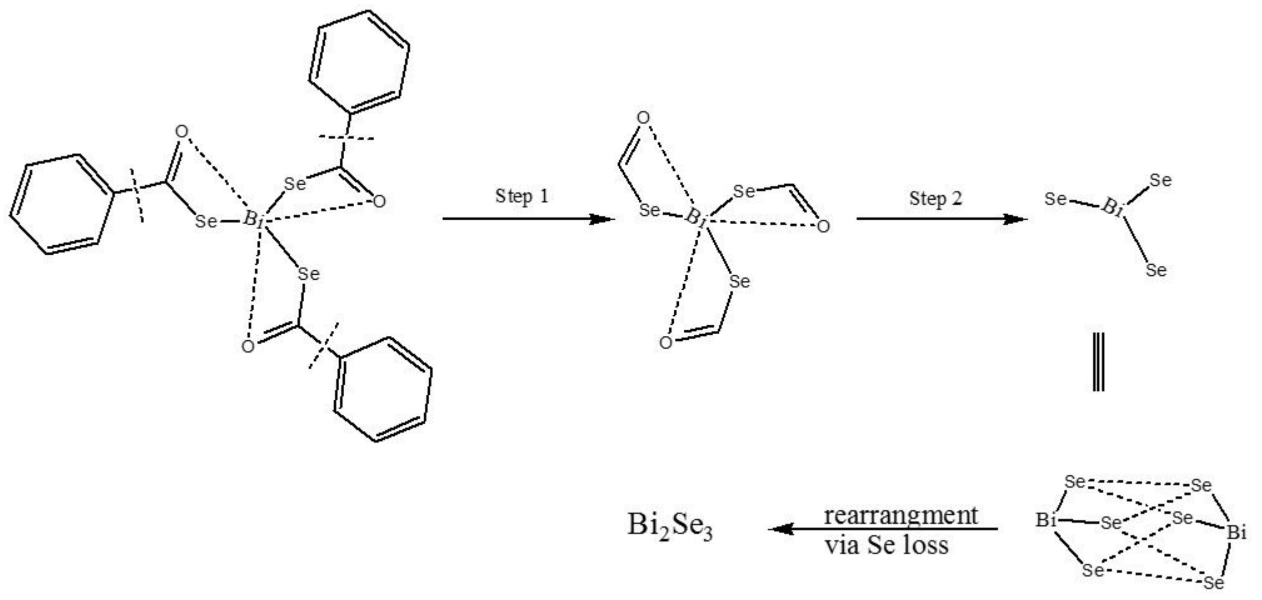

Scheme S1. Stepwise illustration of the proposed decomposition pathway of the *tris*(selenobenzoato)bismuth(III) complex.

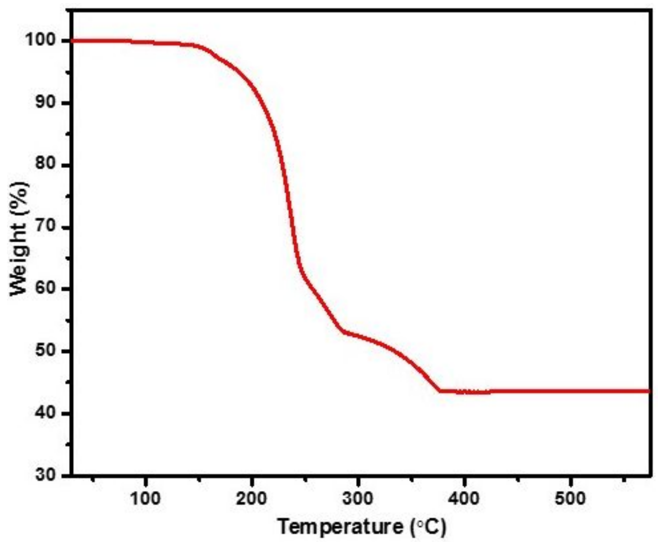

Fig. S1. Thermogravimetric analysis of *tris*(selenobenzoato)bismuth(III) complex, showing three step decomposition.

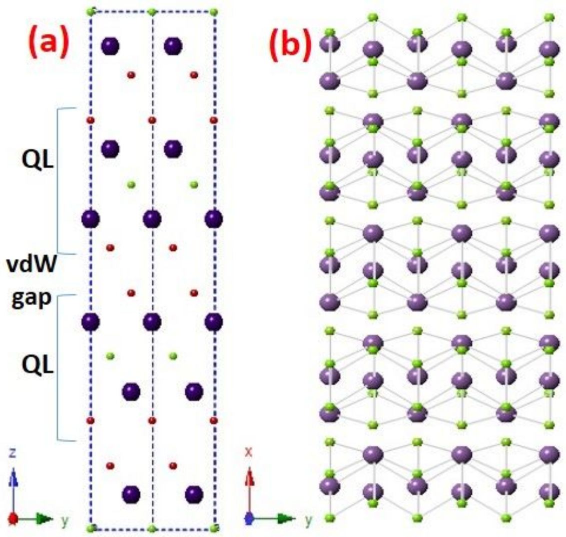

Fig. S2. (a) Bi_2Se_3 crystal structure showing quintuple layer (QL) with Se1-Bi-Se2-Bi-Se1 arrangement and (b) layered structure of Bi_2Se_3.

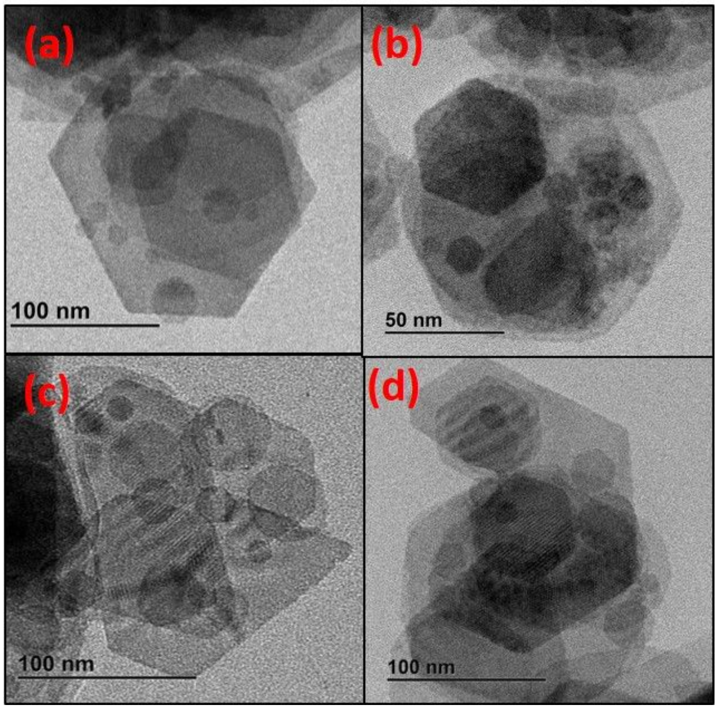

Fig. S3. TEM images showing stacking, buckling and folding of the Bi_2Se_3 nanosheets.

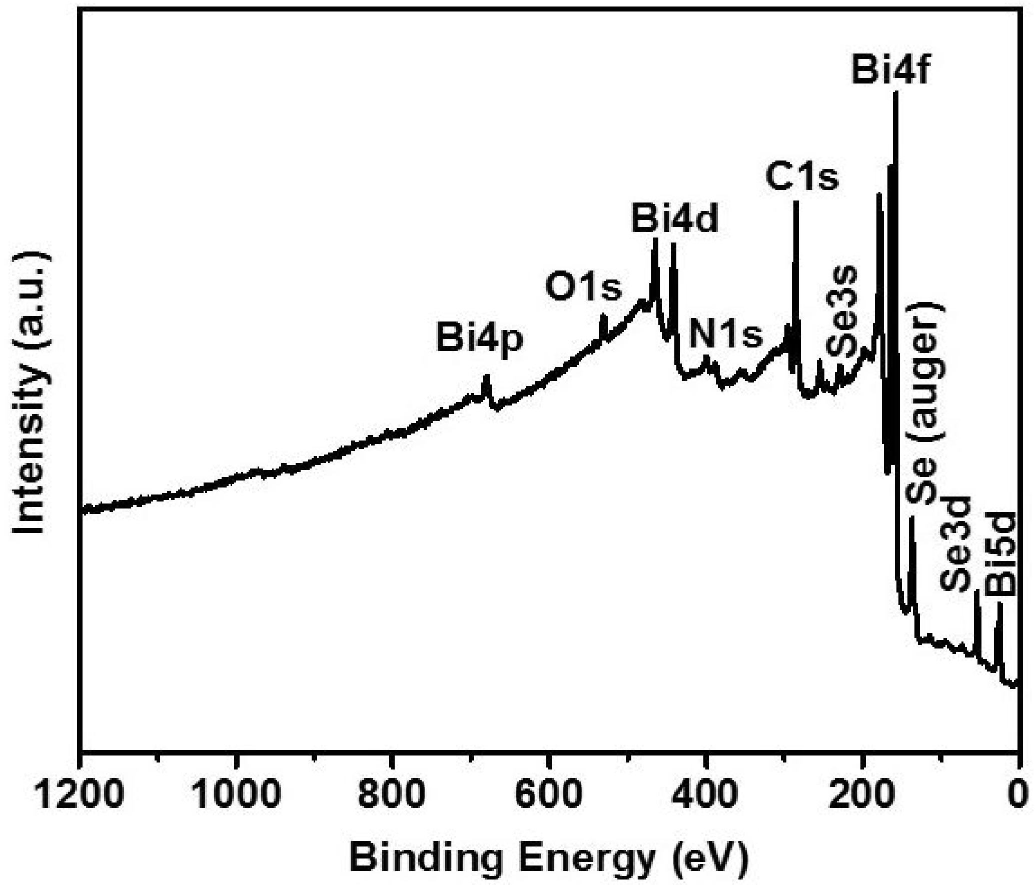

Fig. S4. Survey scan of Bi_2Se_3 nanosheets synthesized in oleylamine.

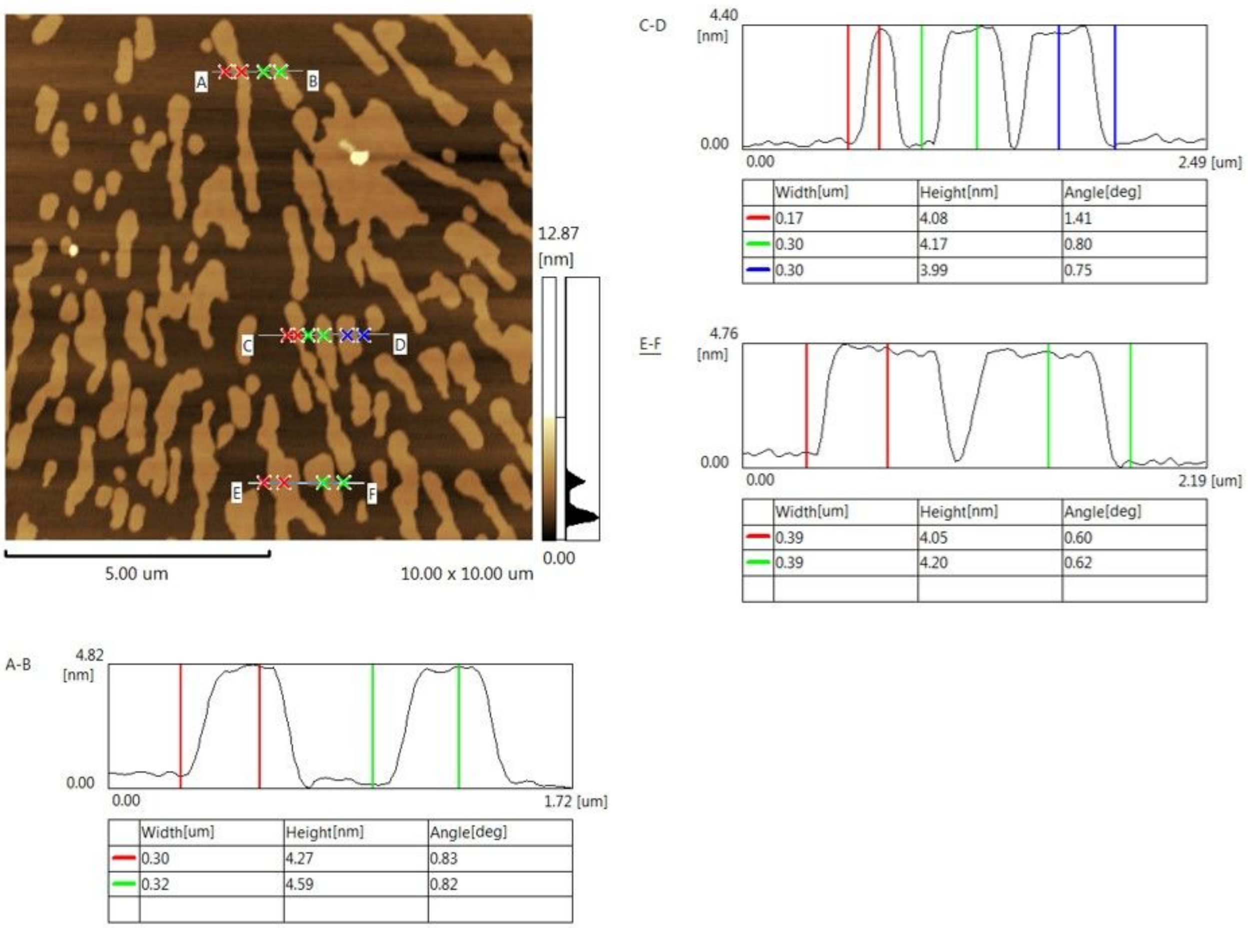

Fig. S5. AFM image showing height profiles of the Bi_2Se_3 nanosheets at different points.

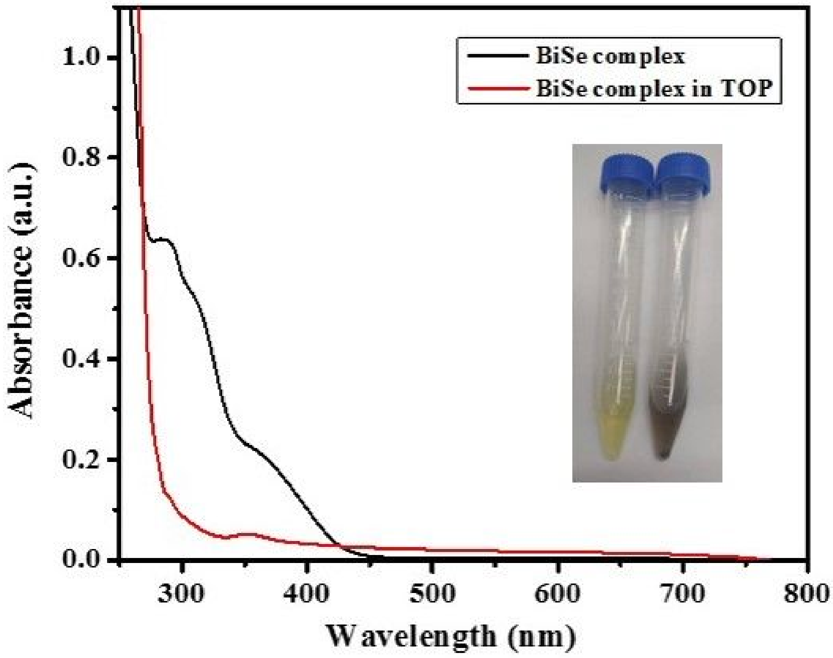

Fig. S6. (a) UV-vis spectrum of bismuth complex dissolved in chloroform, before and after addition of TOP. Inset shows photograph of bismuth complex dissolved in chloroform before (greenish yellow) and after (blackish brown) the addition of TOP.

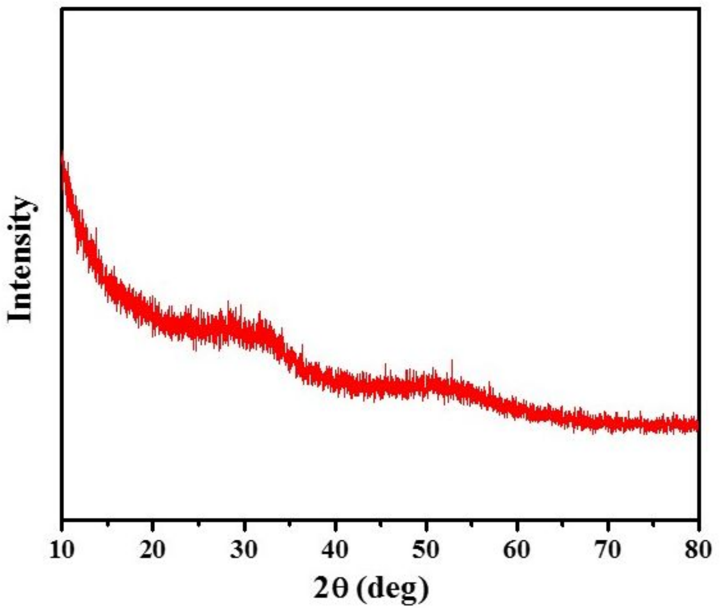

Fig. S7. p-XRD pattern of Bi prepared by decomposition of precursor at room temperature.

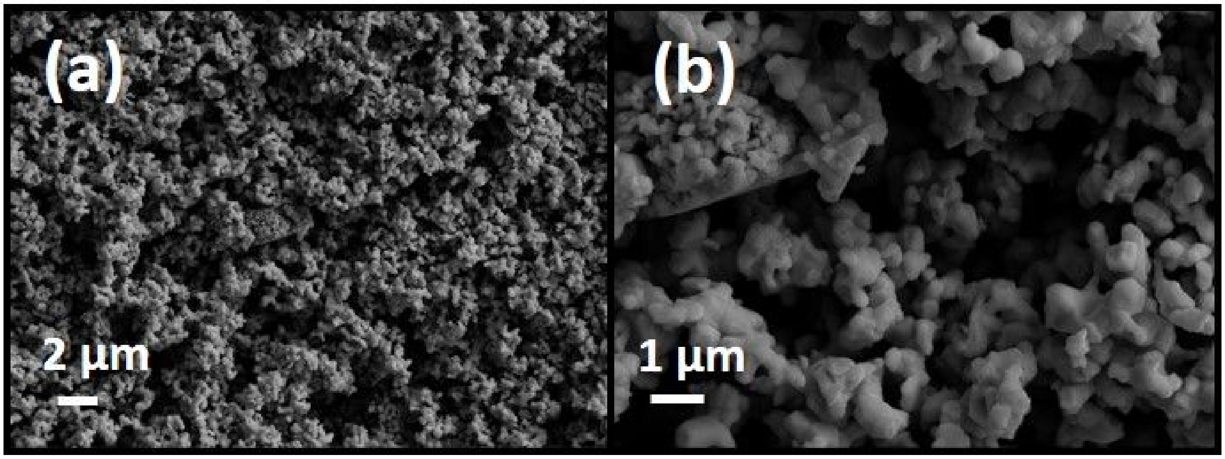

Fig. S8. SEM images of Bismuth at different magnifications.

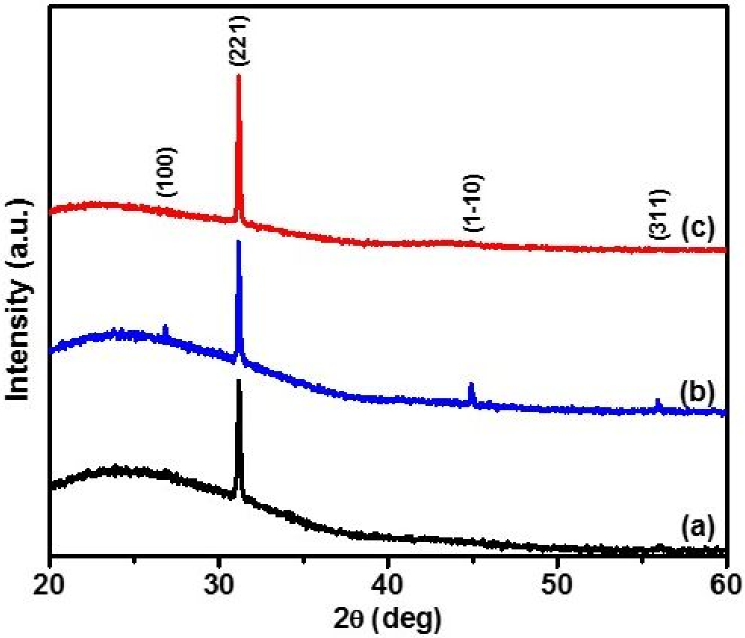

Fig. S9. p-XRD pattern of Bi_2Se_3 thin films deposited at (a) 400 °C, (b) 450 °C and (c) 500 °C.

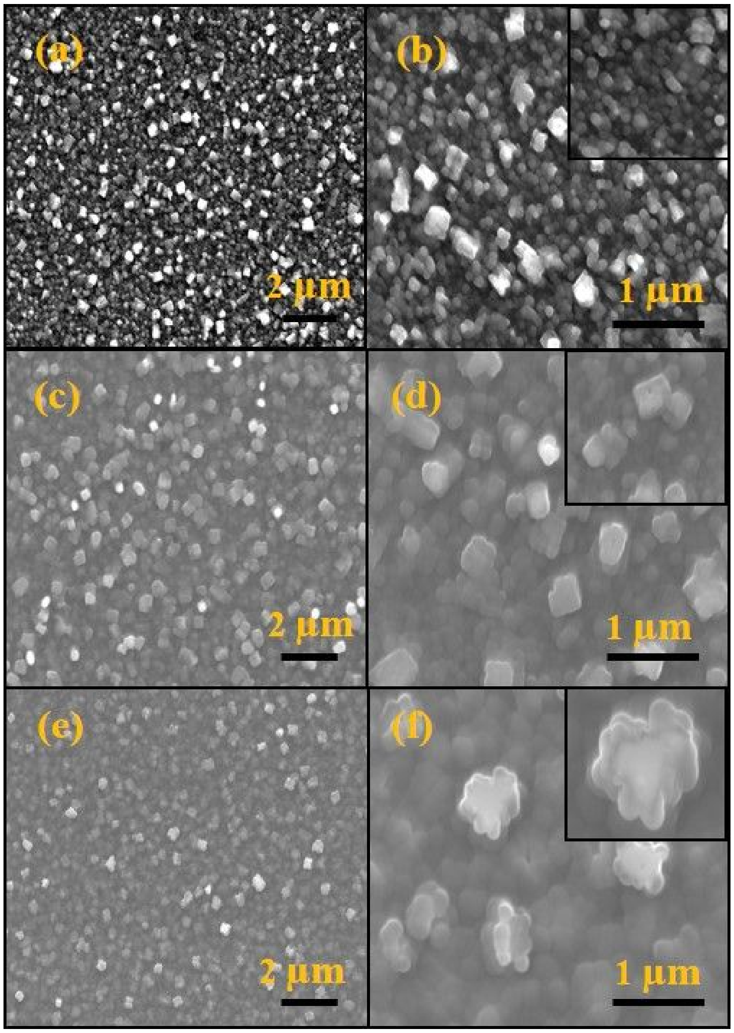

Fig. S10: SEM images of Bi_2Se_3 thin films deposited at (a-b) 400 °C, (c-d) 450 °C and at (e-f) 500 °C.

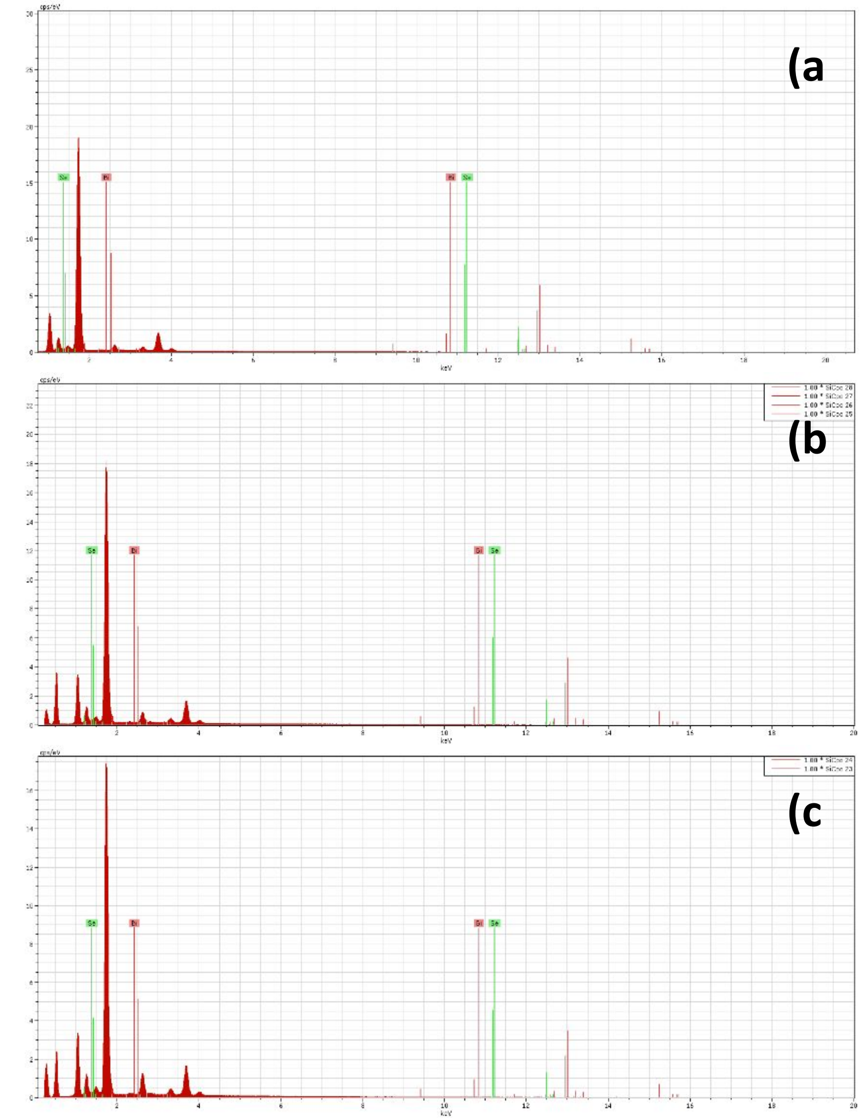

Fig. S11. EDX spectrum of Bi_2Se_3 thin films deposited at (a) 400 °C, (b) 450 °C and (c) 500 °C.

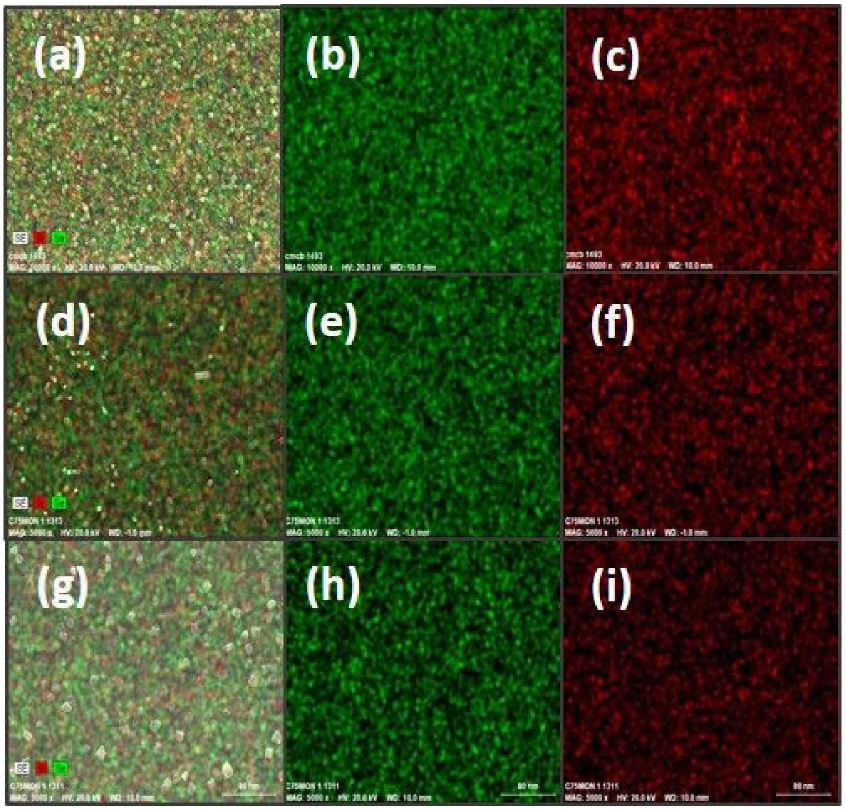

Fig. S12. Elemental mapping of Bi_2Se_3 thin films (bismuth = red, selenium = green) deposited at (a-c) 400 °C, (d-f) 450 °C and (g-i) 500 °C.

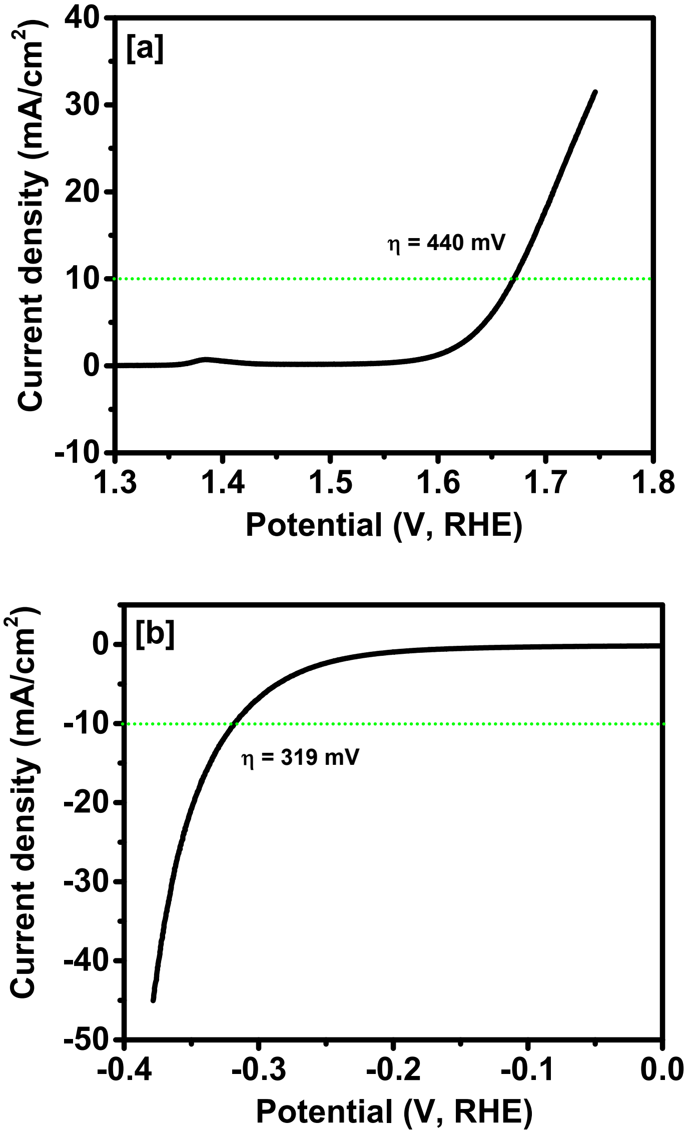

Fig. S13. OER and HER polarization curves for Ni foam.

Table S1. Comparison of OER performance of Bi_2Se_3 nanosheets with previously reported non-precious electrocatalysts.

Catalyst	Overpotential (mV) at 10 mA/cm^2	Tafel slope (mV/dec)	Reference
$MoSe_2$/CdS	860	667	[2]
WSe_2/CdS	730	429	[2]
Mo–N/C@MoS_2	390	72	[3]
Co_3O_4	410	110	[4]
$NiCo_2O_4$ Nanosponge	362	64	[5]
$Co_{0.5}Fe_{0.5}S$@N-MC	410	159	[6]
Zn-Co-S Nanosheets	390	136	[7]
Co_9S_8	602	307	[8]
Co_9S_8/Graphene	441	94	[8]
$AgBiS_2$	414	134	[9]
CoMnP	330	61	[10]
$NiCo_2S_4$	348	89	[11]
NiCo LDH NP	430	62	[12]
Exfoliated Bi_2Se_3	Inactive	-	[13]
**Bi_2Se_3 nanosheets**	**385**	**122**	**This work**
**Bi nanosheets**	**506**	**175**	**This work**

Table S2. Comparison of HER performance of Bi_2Se_3 nanosheets with previously reported non-precious electrocatalysts.

Catalyst	Overpotential (mV) at 10 mA/cm^2	Tafel slope (mV/dec)	Reference
CoS_2	244	133	[14]
Ni_3S_2	310	96	[15]
$NiCo_2S_4$	240	81.3	[15]
NiS	474	124	[16]
NiS_2	454	128	[16]
Ni_2P Nanoparticles	225	100	[17]
$Ni_{0.9}Fe_{0.1}$/NC	231	111	[18]
EG/NiFe-LDH	~380	125	[19]
EG/$Co_{0.85}$Se/NiFe-LDH	260	160	[19]
Zn-Co-S Nanosheets	415	139	[7]
Zn-Co-S Nanoplates	304	131	[7]
CoOx@CN	235	115	[20]
Cobalt-Cobalt Oxide/N-Doped Carbon Hybrids	232	115	[20]
CoP NWs	209	129	[21]
Bi_2Se_3	508	104	[22]
Exfoliated Bi_2Se_3	Inactive	-	[13]

**Bi_2Se_3 nanosheets**	**220**	**178**	**This work**
**Bi nanosheets**	**214**	**158**	**This work**

References

1. A. J. Bard, L. R. Faulkner, J. Leddy and C. G. Zoski, *Electrochemical methods: fundamentals and applications*, Wiley New York, 1980.
2. S. Keltie, *Journal of Materials Chemistry A*, 2018, **6**, 24988-24988.
3. I. S. Amiinu, Z. Pu, X. Liu, K. A. Owusu, H. G. R. Monestel, F. O. Boakye, H. Zhang and S. Mu, *Advanced Functional Materials*, 2017, **27**, 1702300.
4. H. Hu, B. Guan, B. Xia and X. W. Lou, *Journal of the American Chemical Society*, 2015, **137**, 5590-5595.
5. C. Zhu, D. Wen, S. Leubner, M. Oschatz, W. Liu, M. Holzschuh, F. Simon, S. Kaskel and A. Eychmüller, *Chemical Communications*, 2015, **51**, 7851-7854.
6. M. Shen, C. Ruan, Y. Chen, C. Jiang, K. Ai and L. Lu, *ACS Applied Materials & Interfaces*, 2015, **7**, 1207-1218.
7. X. Wu, X. Han, X. Ma, W. Zhang, Y. Deng, C. Zhong and W. Hu, *ACS Applied Materials & Interfaces*, 2017, **9**, 12574-12583.
8. S. Dou, L. Tao, J. Huo, S. Wang and L. Dai, *Energy & Environmental Science*, 2016, **9**, 1320-1326.
9. M. D. Khan, M. Aamir, M. Sohail, S. Bhoyate, M. Hyatt, R. K. Gupta, M. Sher and N. Revaprasadu, *Dalton Transactions*, 2019.
10. D. Li, H. Baydoun, C. u. N. Verani and S. L. Brock, *Journal of the American Chemical Society*, 2016, **138**, 4006-4009.
11. C. Gervas, M. D. Khan, C. Zhang, C. Zhao, R. K. Gupta, E. Carleschi, B. P. Doyle and N. Revaprasadu, *RSC Advances*, 2018, **8**, 24049-24058.
12. H. Liang, F. Meng, M. Cabán-Acevedo, L. Li, A. Forticaux, L. Xiu, Z. Wang and S. Jin, *Nano Letters*, 2015, **15**, 1421-1427.
13. A. Ambrosi, Z. k. Sofer, J. Luxa and M. Pumera, *ACS nano*, 2016, **10**, 11442-11448.
14. H. Zhang, Y. Li, G. Zhang, P. Wan, T. Xu, X. Wu and X. Sun, *Electrochimica Acta*, 2014, **148**, 170-174.
15. A. Sivanantham, P. Ganesan and S. Shanmugam, *Advanced Functional Materials*, 2016, **26**, 4661-4672.
16. N. Jiang, Q. Tang, M. Sheng, B. You, D.-e. Jiang and Y. Sun, *Catalysis Science & Technology*, 2016, **6**, 1077-1084.
17. L. Feng, H. Vrubel, M. Bensimon and X. Hu, *Physical Chemistry Chemical Physics*, 2014, **16**, 5917-5921.
18. X. Zhang, H. Xu, X. Li, Y. Li, T. Yang and Y. Liang, *ACS Catalysis*, 2015, **6**, 580-588.
19. Y. Hou, M. R. Lohe, J. Zhang, S. Liu, X. Zhuang and X. Feng, *Energy & Environmental Science*, 2016, **9**, 478-483.

20. H. Jin, J. Wang, D. Su, Z. Wei, Z. Pang and Y. Wang, *Journal of the American Chemical Society*, 2015, **137**, 2688-2694.
21. J. Tian, Q. Liu, A. M. Asiri and X. Sun, *Journal of the American Chemical Society*, 2014, **136**, 7587-7590.
22. D. Li, J. Lao, C. Jiang, C. Luo, R. Qi, H. Lin, R. Huang, G. I. Waterhouse and H. Peng, *International Journal of Hydrogen Energy*, 2019, **44**, 30876-30884.
